# Functional Immune Cell‐Derived Exosomes Engineered for the Trilogy of Radiotherapy Sensitization

**DOI:** 10.1002/advs.202106031

**Published:** 2022-06-17

**Authors:** Xiaotu Ma, Meinan Yao, Yu Gao, Yale Yue, Yao Li, Tianjiao Zhang, Guangjun Nie, Xiao Zhao, Xiaolong Liang

**Affiliations:** ^1^ Department of Ultrasound Peking University Third Hospital Beijing 100191 P. R. China; ^2^ CAS Key Laboratory for Biomedical Effects of Nanomaterials and Nanosafety & CAS Center for Excellence in Nanoscience National Center for Nanoscience and Technology of China Beijing 100190 P. R. China; ^3^ Beijing Center for Disease Control and Prevention Beijing 100013 P. R. China; ^4^ Key Laboratory of Protein and Peptide Pharmaceuticals CAS Center for Excellence in Biomacromolecules Institute of Biophysics Chinese Academy of Sciences Beijing 100101 P. R. China; ^5^ IGDB‐NCNST Joint Research Center Institute of Genetics and Developmental Biology Chinese Academy of Sciences Beijing 100101 P. R. China

**Keywords:** engineered exosome, immune microenvironment, macrophage polarization, radiotherapy sensitization, tumor hypoxia

## Abstract

The limited efficacy of radiotherapy leads to radio‐resistance and high rates of tumor recurrence and metastasis, which is caused by tumor hypoxia, rapid DNA damage repair, and especially the suppressive immune microenvironment of tumor. Lots of immune cell‐derived exosomes can regulate antitumor immunity, but their application in enhancing radiotherapy is rarely studied. Herein, as a model of concept, M1 macrophage‐derived exosomes (M1Exos) is engineered as effective radiotherapy sensitizers, realizing the trilogy of radiotherapy sensitization: 1) M1Exos is engineered to express catalases on the inside of membrane, which can effectively relieve tumor hypoxia, and enhance DNA damage. 2) The DNA damage repair inhibitor is loaded in M1Exos to effectively inhibit DNA damage repair. 3) M1Exos can polarize M2 macrophages into M1 phenotypes, and the anti‐PD‐L1 nanobody engineered on the outside of M1Exos can relieve the immunosuppression of T cells, both ultimately leading to the remodeling of the tumor suppressive microenvironment. The trilogy of radiotherapy sensitization achieves excellent antitumor efficacy, exhibiting the good utility of engineering immune cell‐derived exosomes as radiotherapy sensitizers, inspiring the future efforts to explore different kinds of immune cell‐derived exosomes for enhanced radiotherapy.

## Graphical abstract

M1 macrophage‐derived exosomes with catalase (CAT) and anti‐PD‐L1 (programmed death ligand‐1) nanobody expressed on membrane and DNA damage repair inhibitor (DDRi) encapsulated inside is engineered as effective radiotherapy sensitizers, realizing the trilogy of radiotherapy sensitization: the relief of tumor hypoxia, the inhibition of DNA damage repair, and the remodeling of tumor suppressive immune microenvironment. This work exhibits the good utility of engineering immune cell‐derived exosomes as effective radiotherapy sensitizers.

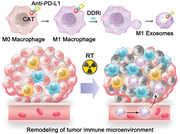

## 1 Introduction

Radiotherapy is one of the most effective treatment modalities for solid tumors.^[^ ^1^, ^2^ ^]^ More than 50% of patients with malignant tumors receive radiotherapy alone or in combination with surgery, chemotherapy, immunotherapy or other treatments.^[^ ^2^, ^3^ ^]^ Radiotherapy is involved in all stages of cancer treatment in the forms of diagnostic radiation, radical radiotherapy, adjuvant/neoadjuvant radiation therapy, and palliative radiation therapy.^[^ ^4^, ^5^ ^]^ Despite the wide clinical application, some intrinsic shortcomings of radiotherapy limit its efficacy: 1) High dose of ionizing radiation causes damage to normal tissue, leading to various adverse effects (e.g., radiation pneumonitis, enteritis, lymphopenia). Although increasing radiation dose can improve the antitumor efficacy, it also increases the radiation damage to normal tissues, i.e., the radiotherapeutic efficacy is limited by its adverse effects.^[^ ^5^ ^]^ 2) Radio‐resistance inherent in many types of cancers (e.g., pancreatic cancer and glioblastoma).^[^ ^6^, ^7^ ^]^ 3) High rates of recurrence and metastasis in many cases largely owing to tumor hypoxia, rapid DNA damage repair, and suppressive immune microenvironment of tumor.^[^ ^5^, ^6^ ^]^ Thus, various radiotherapy sensitizers are exploited to enhance the efficacy and reduce the side effects of radiotherapy, mainly by increasing DNA damage of radiotherapy and therefore reducing the required radiation dose.^[^ ^8^, ^9^, ^10^ ^]^ However, the already developed radiotherapy sensitizers cannot fully satisfy clinical needs.^[^ ^9^ ^]^ For instance, chemotherapeutic drugs with function of impairing DNA damage repair can sensitizing radiotherapy, but often cause severe side effects (e.g., renal toxicity of cis‐platinum);^[^ ^11^ ^]^ the most promising radiotherapy sensitizers, the derivatives of nitroimidazole (e.g., nimorazole), achieve the fixation of the oxidative damage derived from radiotherapy, but are still under clinic trials;^[^ ^12^ ^]^ high atomic number nanomaterials (e.g., gold, gadolinium) boost the generation of reactive oxygen species, but suffer from poor degradability and long‐term in vivo safety concern;^[^ ^8^, ^13^ ^]^ hyperbaric oxygen supply for directly reliving tumor hypoxia has dangerous complications (e.g., barotrauma and hypoxic seizures).^[^ ^14^ ^]^ Therefore, there is an urgent need to develop safe and effective radiotherapy sensitizers.^[^ ^9^ ^]^

Exosomes are membrane vesicles secreted by most cells with size of 30–150 nm.^[^ ^15^, ^16^ ^]^ Exosomes are rich in a variety of active molecules (e.g., nucleic acids, proteins, lipids, metabolites), and can transport these molecules from donor cells to recipient cells through various transport mechanisms, including endocytosis or phagocytosis, receptor‐ligand interaction, or direct membrane fusion.^[^ ^15^, ^17^ ^]^ The exosome‐mediated cell‐to‐cell communication is ubiquitous in the biological world and plays important roles in many pathological/physiological processes.^[^ ^17^ ^]^ Inspired by the ability to transport molecules between cells, engineered exosomes are being explored as drug delivery systems.^[^ ^15^ ^]^ Besides the cell‐to‐cell transportability, engineered exosomes have many advantages compared with traditional artificial drug delivery systems, including excellent biocompatibility, superb transcellular permeability, low toxicity and immunogenicity, easier escape from the clearance of the immune system, natural stability in circulation and complete degradability in vivo.^[^ ^18^, ^19^ ^]^ Exosomes contain many active components of donor cells, endowing them with many special functions.^[^ ^15^, ^16^ ^]^ For instance, tumor cell‐derived exosomes inherit the membrane protein profile of donor cells, and can be actively targeted to the homologous tumor tissue.^[^ ^15^, ^20^ ^]^

Owing to these advantages, engineered exosomes have been widely utilized as the delivery systems of chemotherapeutic/immunotherapeutic drugs, therapeutic nucleic acids, and photothermal/photodynamic agents.^[^ ^15^, ^16^ ^]^ Compared with these abundant studies, the application for delivering radiotherapy sensitizers is insufficiently studied. The reported studies involve employing exosomes for the delivery of bionic prodrugs or manganese carbonyl to enhance radiotherapy.^[^ ^21^ ^]^ Only tumor cell‐derived exosomes are used in these studies for improving tumor‐targeting ability of radiotherapy sensitizers. However, except from improving tumor‐targeting ability, the function of tumor‐cell derived exosomes is relatively monotonous. Immune‐derived exosomes contain lots of cargos that function in immune regulation, gaining more and more attention.^[^ ^19^ ^]^ For instance, dendritic‐cell‐derived exosomes with the peptide‐major histocompatibility complex (MHC) complex on surface can active T cells to promote antitumor immune;^[^ ^22^ ^]^ natural killer cell‐derived exosomes display Fas ligand and perforin molecules to induce antitumor cytotoxicity.^[^ ^23^ ^]^ Whether it is possible to use immune cell‐derived exosomes as the delivery system of radiotherapy sensitizers, thus enhancing radiotherapy by immune regulation? It has not been explored to date.

Macrophage is a type of immune cells, which can be categorized into pro‐tumor M2 phenotypes and anti‐tumor M1 phenotypes.^[^ ^24^ ^]^ It has been revealed that artificial nanovesicles derived from M1 macrophage membrane can accumulate in tumor region, re‐polarize M2 TAMs (tumor‐infiltrated macrophages) into M1 phenotypes.^[^ ^25^, ^26^ ^]^ Meanwhile, previous studies also reveal that radiotherapy leads to the up‐regulation of immunosuppressive cells, including the promotion of M2 macrophage phenotype, which can inhibit T cell‐mediated anti‐tumor immune response and promote tumor progression.^[^ ^6^, ^27^ ^]^ Therefore, M1 macrophage‐derived exosomes (M1Exos) could possibly enhance the efficacy of radiotherapy.

**Scheme 1:**
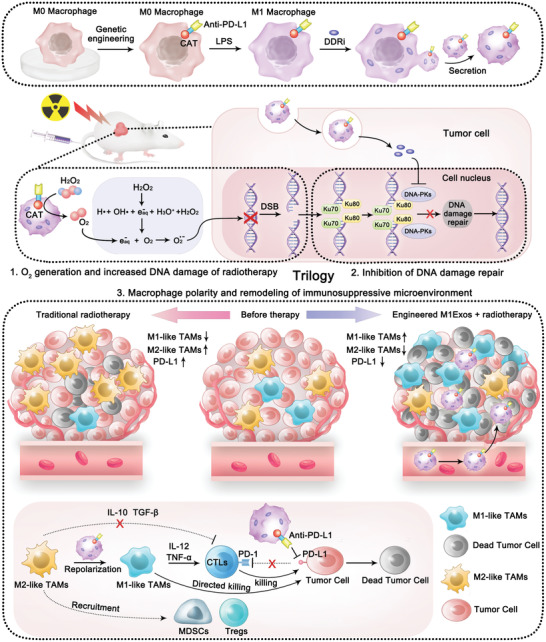
Schematic illustration of employing engineered M1Exos for the trilogy of radiotherapy sensitization: The catalases expressed on the inner side of M1Exos membrane could effectively relieve tumor hypoxia and enhance DNA damage; DDR inhibitors loaded in M1Exos could effectively inhibit DDR; M1Exos could polarize M2 macrophages into M1 phenotypes, and the anti‐PD‐L1 nanobody (Anti‐PD‐L1) expressed on the outer side of M1Exos membrane could relieve the immunosuppression of T cells, both ultimately leading to the remodeling of the tumor suppressive microenvironment. CAT, catalase; LPS, lipopolysaccharide; DDRi, DNA damage repair inhibitor; DSB, DNA double‐strand break; DNA‐PKs, DNA‐dependent protein kinases.

Besides the tumor immunosuppressive microenvironment caused by radiotherapy, the efficacy of radiotherapy is greatly limited by the hypoxia of solid tumors,^[^ ^28^, ^29^, ^30^ ^]^ and the rapid repair of DNA damage after radiotherapy.^[^ ^30^, ^31^, ^32^ ^]^ Herein, as a model of concept, M1 macrophage‐derived exosomes (M1Exos) with catalases and anti‐PD‐L1 (programmed death ligand‐1) nanobody expressed on surface was engineered for the delivery of radiotherapy sensitizers (**Scheme** **1**), aiming to solve the above three limitations simultaneously, realizing the trilogy of radiotherapy sensitization: 1) The antitumor efficacy of radiotherapy relies on DNA damage induced by high‐energy X‐ray or *γ*‐rays.^[^ ^2^ ^]^ Hypoxia is the common hallmark of solid tumors, and directly affects the degree of DNA damage of radiotherapy.^[^ ^9^, ^28^, ^33^ ^]^ The catalase and its mimics have been used to relieve tumor hypoxia and enhance radiotherapy by catalyzing endogenous H_2_O_2_ in tumor.^[^ ^34^ ^]^ Herein, the catalase expressed on the inner membrane of engineered M1Exos could effectively relieve tumor hypoxia and enhance DNA damage. 2) Tumor cells initiate DNA damage repair (DDR) immediately after radiotherapy. DDR inhibitors loaded in engineered M1Exos could effectively inhibit DDR, leading to the increased cell death. Although radiation induces various forms of DNA damage, and produces more base lesions and DNA single‐strand breaks (SSBs), the DNA double‐strand breaks (DSBs) are the most lethal to tumor cells. The repair of DSBs occurs mainly through the nonhomologous end‐joining pathway, and requires several factors among which the DNA‐dependent protein kinase (DNA‐PK) represents the cornerstone of the DSBs repair complex.^[^ ^35^ ^]^ Nedisertib was chosen as the DDR inhibitor due to its low half maximal inhibitory concentration (IC_50_, < 3 × 10^−9^ m) and high specificity for inhibiting DNA‐PK. 3) Cell death causes the inflammatory microenvironment of tumor, promoting lymphocyte infiltration,^[^ ^32^ ^]^ but also up‐regulates the immunosuppressive cells (e.g., M2 TAMs) ^[^ ^6^, ^27^ ^]^ and receptors (PD‐L1),^[^ ^36^ ^]^ leading to the tumor immunosuppressive microenvironment.^[^ ^6^ ^]^ Engineered M1Exos could polarize M2 TAMs into M1 phenotypes, and the anti‐PD‐L1 nanobody expressed on the outer membrane of engineered M1Exos could relieve the immunosuppression of T cells, ultimately leading to the remodeling of the tumor immune microenvironment and the improvement of radiotherapeutic efficacy.

### 1.1 Fabrication and Characterization of Engineered M1Exos

**Figure 1:**
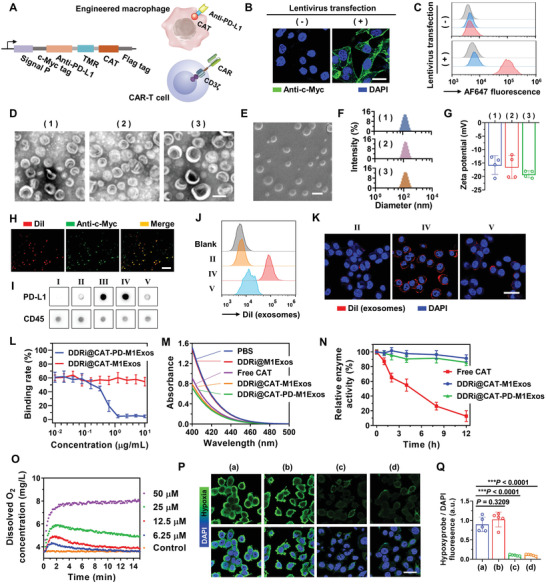
Fabrication and characterization of engineered M1Exos. A) The gene structure of lentiviral vector coding the fusion protein CAT‐TMR‐Anti‐PD‐L1‐c‐Myc, and its expression pattern on cell membrane of RAW 264.7 cells. Anti‐PD‐L1‐c‐Myc was located on the outside of cell membrane, and CAT was on the inside. This structure was inspired by Car‐T cell. TMR, transmembrane region. Signal P, signal peptide. B) The expression of the fusion protein in RAW 264.7 was examined by immunofluorescence staining of c‐Myc tag. The fusion protein could be successfully expressed on cell membrane after stable transfection of lentivirus (+). Nucleus was stained with DAPI. Scale bar, 20 µm. C) The stable expression of fusion protein on cell membrane of RAW 264.7 was confirmed by flow cytometry. Gray, blank RAW 264.7 cells without staining; blue, cells were stained with Alexa Fluor 647 (AF647)‐conjugated anti‐rabbit secondary antibodies; red, cells were first stained with rabbit anti‐c‐Myc antibodies, followed by the staining of secondary antibodies. D) TEM images of M1Exos (1), CAT‐PD‐M1Exos (2), and DDRi@CAT‐PD‐M1Exos (3). Scale bar, 100 µm. E) SEM images of DDRi@CAT‐PD‐M1Exos. Scale bar, 100 µm. F) The hydrodynamic diameter distribution and G) zeta potentials of 1) M1Exos, 2) CAT‐PD‐M1Exos, and 3) DDRi@CAT‐PD‐M1Exos. H) The co‐localization of the anti‐c‐Myc antibody‐stained fusion protein and the DiI‐labeled DDRi@CAT‐PD‐M1Exos by CLSM, confirming the existence of fusion protein on DDRi@CAT‐PD‐M1Exos. Scale bar, 1.0 µm. I) The affinity between Anti‐PD‐L1 on DDRi@CAT‐PD‐M1Exos and mouse PD‐L1 protein was verified by dot blotting. PD‐L1 protein was adsorbed on nitrocellulose membrane, followed by the addition of PBS (I), DDRi@CAT‐M1Exos (II), CAT‐PD‐M1Exos (III), DDRi@CAT‐PD‐M1Exos (IV), DDRi@CAT‐PD‐M1Exos plus excess amount of Anti‐PD‐L1 (V), and the binding of different M1Exos onto membrane was examined by HRP (horse radish peroxidase)‐conjugated anti‐c‐Myc. The expression of CD45 on M1Exos was examined as input, and the equal amounts of different M1Exos (0.5 µg) were adsorbed onto membrane, followed by the addition of HRP‐conjugated antimouse CD45. J,K) The affinity between Anti‐PD‐L1 (on DDRi@CAT‐PD‐M1Exos) and PD‐L1 (on LLC cells) was verified by J) flow cytometry and K) CLSM. LLC cells were incubated with DiI‐labeled CAT‐M1Exos (II), DDRi@CAT‐PD‐M1Exos (IV), and DDRi@CAT‐PD‐M1Exos plus excess amount of Anti‐PD‐L1 (V). Nucleus was stained with DAPI. Scale bar, 30 µm. (L) The competition binding assay for confirming the affinity of Anti‐PD‐L1 on DDRi@CAT‐PD‐M1Exos. LLC cells were co‐incubated with a fixed concentration of Cy5.5‐conjugated Anit‐PD‐L1 along with gradient concentrations of DDRi@CAT‐M1Exos or DDRi@CAT‐PD‐M1Exos. The concentration of un‐binding Cy5.5‐Anit‐PD‐L1 was measured by fluorescence intensity of Cy5.5. (M) Confirmation of enzyme activity of CAT in DDRi@CAT‐PD‐M1Exos. The concentration of H_2_O_2_ was measured by the ammonium molybdate method, and the decrease of absorbance at 405 nm indicating the decomposition of H_2_O_2_. (N) The relative enzyme activity of CAT after incubating free CAT, DDRi@CAT‐M1Exos or DDRi@CAT‐PD‐M1Exos with proteinase K for different time (0–12 h). The membrane of M1Exos could protect CAT from hydrolysis. O) DDRi@CAT‐PD‐M1Exos was mixed with different concentration of H_2_O_2_ (6.25–50 × 10^−6^ m), and the concentration of dissolved oxygen was measured by on‐line oxygen dissolving meter. The control group was 50 × 10^−6^ m H_2_O_2_ without the addition of DDRi@CAT‐PD‐M1Exos. P,Q) Hypoxia level of LLC cells after incubating cells with a) PBS, b) DDRi@M1Exos, c) DDRi@CAT‐PD‐M1Exos and d) free CAT. Cells were maintained in hypoxia condition with 10 × 10^−6^ m H_2_O_2_. Cellular hypoxia was stained with the hypoxyprobe‐1 kit, and nucleus was stained with DAPI. Data are presented as mean ± SD (*n* = 5, *p < 0.05, **p < 0.01, ***p < 0.001).

Using lentiviral transfection, the exogenous fusion protein CAT‐TMR‐Anti‐PD‐L1‐c‐Myc was stable expressed on the membrane of RAW 264.7 cells (a mouse macrophage cell line). The domain of anti‐PD‐L1 nanobody (Anti‐PD‐L1) and c‐Myc tag was located on the outside of cell membrane, and the domain of catalase (CAT) was on the inside (**Figure** **1**A). Anti‐PD‐L1 and CAT were linked by the transmembrane region (TMR). The special structure of fusion protein was inspired by chimeric antigen receptor (CAR)‐T cell, which has CAR and CD3*ζ* region located on the outside and inside of cell membrane (Figure 1A). Confocal laser scanning microscopy (CLSM) images of the immunofluorescence staining of c‐Myc tag confirmed the successful expression of the fusion protein in the cytomembrane of RAW 264.7 cells (Figure 1B). Flow cytometry analysis confirmed the c‐Myc tag was located on the outside of cell membrane (Figure 1C).

RAW 264.7 cells were polarized into M1 phenotypes by lipopolysaccharide (LPS), and the secreted exosomes (M1Exos) were obtained by filtration and ultracentrifugation. M1Exos with the expression of CAT and Anti‐PD‐L1 (CAT‐PD‐M1Exos) were obtained from genetically engineered RAW 264.7 cells. DDRi‐loaded CAT‐PD‐M1Exos (DDRi@CAT‐PD‐M1Exos) was further obtained from genetically engineered RAW 264.7 cells with the uptake of DDRi. The content of CAT in engineered M1Exos was 2.74% (0.0274 mg mg^−1^) determined by enzyme‐linked immunosorbent assay (ELISA) kit for CAT (the amount of M1Exos was defined as the amount of total protein), and the content of DDRi in engineered M1Exos was 16.62 nmol mg^−1^ determined by HPLC‐MS. The images of transmission electron microscope (TEM) (Figure 1D), freeze‐etching scanning electron microscope (SEM) (Figure 1E) exhibited M1Exos, CAT‐PD‐M1Exos, and DDRi@CAT‐PD‐M1Exos had spherical and uniform morphology. There were similar in hydrodynamic diameter distributions (≈140 nm) (Figure 1F) and zeta potentials (≈−15 mV) (Figure 1G). These exosomes exhibited good stability. The average diameter, polydispersity, and zeta potential remained changeless after the storage at 4 °C for 14 d (Figure S1, Supporting Information), or after the three times of cycles of freezing and thawing (Figure S2, Supporting Information). The stability and catalase enzyme activity of engineered M1Exos was examined after the treatment of different temperature (room temperature, 37, 42 °C) and pH values (pH = 5.4) for 24 h, and examined after the treatment of different dose of X‐ray radiation (2 Gy, 6 Gy). These treatments had little influence on the average diameter, polydispersity, and zeta potential of DDRi@CAT‐PD‐M1Exos (Figures S3–S5, Supporting Information), and the treatment of acidic pH and radiation also had little influence the catalase activity of exosomes (Figures S4 and S5, Supporting Information), further demonstrating the good stability of engineered exosomes. As naturally derived nanoexosomes, M1Exos had excellent biocompatibility, and exhibited no cytotoxicity to different cell lines in vitro (Figure S6, Supporting Information).

To confirm the fusion protein was expressed in the engineered exosomes, 1,1'‐dioctadecyl‐3,3,3',3'‐tetramethylindocarbocyanine perchlorate (DiI)‐labeled DDRi@CAT‐PD‐M1Exos were stained with anti‐c‐Myc antibodies. CLSM images exhibited the co‐localization of c‐Myc tag and DiI‐labeled cell membrane (Figure 1H), demonstrating the existence of fusion protein in exosome membrane. The affinity of Anti‐PD‐L1 on DDRi@CAT‐PD‐M1Exos was detailedly studied. Dot blotting assay demonstrated the Anti‐PD‐L1 on exosomes had high specific affinity to PD‐L1 protein (Figure 1I). PD‐L1 protein was adsorbed on nitrocellulose membrane, followed by the addition of DDRi@CAT‐M1Exos, DDRi@CAT‐PD‐M1Exos, or DDRi@CAT‐PD‐M1Exos + Anti‐PD‐L1. Obviously higher amount of DDRi@CAT‐PD‐M1Exos could bind to PD‐L1 compared with DDRi@CAT‐M1Exos, and this binding behavior could be blocked by the excess amount of Anti‐PD‐L1. Mouse Lewis carcinoma (LLC) cells were incubated with DiI‐labeled exosomes, flow cytometry analysis (Figure 1J) and CLSM images (Figure 1K) exhibited DDRi@CAT‐PD‐M1Exos could bind to LLC cells, and the binding could be blocked by excess amount of Anti‐PD‐L1. The competition binding assay further confirmed the affinity of Anti‐PD‐L1 on exosomes (Figure 1L). LLC cells were co‐incubated with a fixed concentration of Cy5.5‐conjugated Anit‐PD‐L1 along with serial dilutions of DDRi@CAT‐M1Exos or DDRi@CAT‐PD‐M1Exos. Only the DDRi@CAT‐PD‐M1Exos could block the binding of Cy5.5‐Anit‐PD‐L1 onto LLC cells (Figure 1L), exhibiting the specific affinity of Anti‐PD‐L1.

The enzyme activity of CAT in DDRi@CAT‐PD‐M1Exos and the ability of reliving hypoxia were examined. Only DDRi@CAT‐M1Exos and DDRi@CAT‐PD‐M1Exos could catalyze the hydrolysis of H_2_O_2_ rather than DDRi@M1Exos (Figure 1M), exhibiting the enzyme activity of CAT in engineered M1Exos. Upon the treatment of proteinase K for 12 h, the enzyme activity of DDRi@CAT‐M1Exos or DDRi@CAT‐PD‐M1Exos only had a slight decrease (< 15%) (Figure 1N). To the stark contrast, the enzyme activity of free CAT had a great decrease (> 85%). Therefore, cell membrane of exosomes could protect CAT from enzymolysis, which could increase the stability of CAT in blood circulation. To examine the ability of generating O_2_, M1Exos, DDRi@M1Exos, DDRi@PD‐M1Exos, and DDRi@CAT‐PD‐M1Exos was mixed with H_2_O_2_ solution, the concentration of dissolved oxygen was measured by on‐line oxygen dissolving meter. The concentration of dissolved oxygen had little change when mixing M1Exos, DDRi@M1Exos, or DDRi@PD‐M1Exos with H_2_O_2_ (Figure S7, Supporting Information). However, the concentration of dissolved oxygen was largely increased when mixing DDRi@CAT‐PD‐M1Exos with H_2_O_2_. Therefore, CAT in DDRi@CAT‐PD‐M1Exos could catalyze the hydrolysis of H_2_O_2_ to generate O_2_. The ability of relieving cellular hypoxia was further examined. Mouse Lewis carcinoma (LLC) cells were maintained in 1 vol% O_2_ for 24 h to induce cells into hypoxic status. Both DDRi@CAT‐PD‐M1Exos and free CAT could efficiently relieve the hypoxic status of LLC cells, while DDRi@M1Exos without the expression of CAT had little influence on the hypoxic status (Figure 1P,Q). The H_2_O_2_ concentrations in tumor cells and solid tumors were examined both in vitro and in vivo. The intracellular H_2_O_2_ concentrations in tumor cell lines (LLC, A549, 4T1 cells) ranged from 17.26–41.45 µmol g^−1^ protein, which were much higher than that of normal cell line HUVECs (Human umbilical vein vessel endothelial cells, 0.52 µmol g^−1^ protein, Figure S8A, Supporting Information). The H_2_O_2_ concentrations in culture medium of tumor cell lines cultured for 48 h ranged from 0.64−2.40 × 10^−6^ m, which were also much higher than that of HUVECs (0.06 × 10^−6^ m, Figure S8B, Supporting Information). The average H_2_O_2_ concentration in LLC tumors (≈160 mm^3^) was 2249.34 nmol g^−1^, and the H_2_O_2_ concentration in major organs ranged from 0.67‐6.36 nmol g^−1^ (Figure S8C, Supporting Information). Therefore, the H_2_O_2_ concentrations in LLC tumor was 350–3400 folds as high as that of normal tissue.

### 1.2 In Vitro Radiotherapy Sensitization Effect of Engineered M1Exos

**Figure 2:**
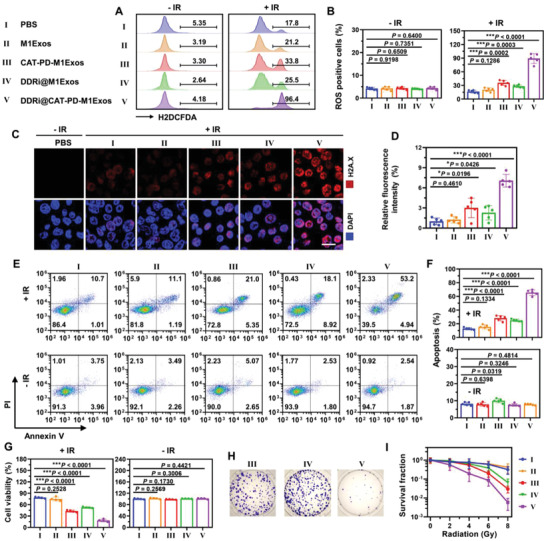
In vitro radiotherapy sensitization effect of engineered M1Exos. LLC cells was maintained in hypoxia condition (1 vol% O_2_) with 10 × 10^−6^ m H_2_O_2_. After the addition of N_2_‐saturated PBS (I) or exosomes (II‐V) at 2 µg mL^−1^ (the concentrations of total proteins in exosomes determined by BCA protein quantification method), cells were treated with 2 Gy X‐ray irradiation. A,B) Flow cytometry analysis of the intracellular ROS level of LLC cells with irradiation (−IR) or without irradiation (+IR). The ROS level was measured by the fluorescence of H2DCFDA. Data are presented as mean ± SD (*n* = 5, *p < 0.05, **p < 0.01, ***p < 0.001). C,D) The level of DNA double strand break of LLC cells after irradiation was examined by the staining of *γ*‐H2A.X. Nucleus was stained with DAPI. Scale bar, 20 µm. E,F) Flow cytometry analysis of the percentage of apoptotic LLC cells with/without irradiation. The apoptotic cells were examined by Annexin V‐FITC/PI double staining. The sum of Annexin V‐positive and PI‐positive cells in total cells were calculated as the percentage of apoptotic cells. G) Cell viability of LLC cells with/without irradiation was examined by CCK‐8 assay. Data are presented as mean ± SD (*n* = 4, *p < 0.05, **p < 0.01, ***p < 0.001). H,I) Colony formation assay after treating LLC cells with different agents (I–V) and irradiation. Photograph of G) cell colonies and H) survival fraction of LLC cells after different doses of irradiation was shown (0–8 Gy). Data are presented as mean ± SD (*n* = 5, *p < 0.05, **p < 0.01, ***p < 0.001).

The radiotherapy sensitization of DDRi@CAT‐PD‐M1Exos was derived from two aspects: (1) CAT could relieve hypoxia, therefore increasing the generation of reactive oxygen species (ROS) during radiotherapy, and enhancing the DNA damage of tumor cells. (2) DDRi could inhibit the repair of DNA damage, leading to the death of tumor cells. For evaluating the radiotherapy sensitization effect of engineered M1Exos, a relatively low dose (2 Gy) of X‐ray irradiation was adopted. The ROS generation of engineered M1Exos was first explored. Upon the treatment of 2 Gy X‐ray irradiation (IR), the ROS level of LLC cells increased by 2.97 folds compared with before irradiation [Group I + IR versus Group I ‐IR, 16.57% versus 4.17% (H2DCFDA‐positive cells)] (**Figure** **2**A,B). To the contrast, the treatment of CAT‐PD‐M1Exos + IR largely increased ROS level by 7.20 folds (Group III + IR versus Group III ‐IR, 35.51% versus 4.33%). DDRi@M1Exos + IR could only slightly increase the generation of ROS. However, DDRi@CAT‐PD‐M1Exos dramatically increase the generation of ROS by as high as 19.48 folds due to enhanced cell damage (Group V + IR versus Group V ‐IR, 89.08% versus 4.35%). DNA double‐strand break (DSB) was the main cause of cell death. The ability of engineered M1Exos to increase the level of DSB was examined. M1Exos + IR had little influence on DSB compared with PBS + IR (Figure 2C,D). However, both CAT‐PD‐M1Exos + IR and DDRi@M1Exos + IR could significantly increase the level of DSB compared with PBS + IR. Much greater elevation in DSB generation was achieved by DDRi@CAT‐PD‐M1Exos + IR, which was increased by 6.06 folds compared with PBS + IR. Comet assay was also employed for analyze the level of DNA damage after 1 Gy X‐ray radiation. The tail moment of comet assay combines tail length and tail intensity in one single value, which is the most useful and frequently used parameter for analyzing DNA damage.^[^ ^37^ ^]^ As shown in Figure S9 (Supporting Information), the average tail moment of CAT‐PD‐M1Exos + IR and DDRi@M1Exos + IR was 10.35 (*n* = 45) and 9.43 (*n* = 32), which were both significantly higher than that of PBS + IR (3.92, *n* = 51), demonstrating the increased DNA damage due to the relief of hypoxia and the inhibition of DNA damage repair, respectively. DDRi@CAT‐PD‐M1Exos + IR achieve the highest tail moment (21.73, *n* = 53), which was resulted from the combination of the radiotherapy sensitization effect of CAT and DDRi.

The assay of cell apoptosis, cell viability, and colony formation was respectively carried out for the evaluation of radiotherapy sensitization. Cell apoptosis was examined by flow cytometry analysis of Annexin V/PI double staining, and cell viability was examined by CCK‐8 assay to reflect cellular metabolic activity. The reproductive capacity of tumor cells was evaluated by colony formation assay, which was the gold standard for evaluating radiotherapy sensitization. Compared with PBS + IR, both CAT‐PD‐M1Exos + IR and DDRi@M1Exos + IR could significantly increase the level of cell apoptosis (Figure 2E,F), and decrease cell viability (Figure 2G) and clonogenic survival fraction (Figure 2H,I). Compared with CAT‐PD‐M1Exos + IR and DDRi@M1Exos + IR, much greater elevation in the radiotherapy sensitization effect was achieved by DDRi@CAT‐PD‐M1Exos + IR. Taken together, by the synergistic effect of CAT and DDRi, engineered M1Exos could largely increase the generation of ROS, enhance the level of DSB, leading to obviously increased cell death and decreased reproductive capacity, exhibiting strong radiotherapy sensitization effect. Meanwhile, DDRi@CAT‐PD‐M1Exos without IR had little influence on cell metabolism owing to the good biocompatibility. The radiotherapy sensitization effect of engineered M1Exos was also examined on the mouse breast cancer 4T1 cells. Strong radiotherapy sensitization effect of DDRi@CAT‐PD‐M1Exos was confirmed by the significant increase of cell apoptosis (Figure S10, Supporting Information), the largely decrease of cell viability (Figure S11, Supporting Information) and clonogenic survival fraction (Figure S12, Supporting Information).

### 1.3 The polarization of macrophages regulated by engineered M1Exos

**Figure 3:**
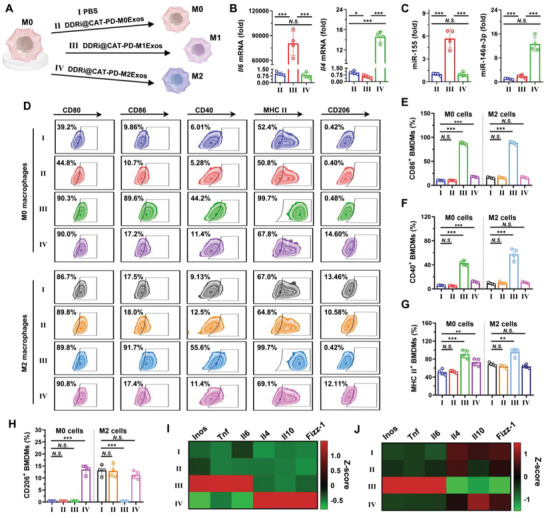
The polarization of macrophages regulated by engineered M1Exos. A) Schematic diagram of macrophage polarization. M0 macrophages could be polarized to M1 phenotypes by engineered M1Exos, but polarized to M2 phenotypes by M2Exos. The relative expression level of B) mRNA and C) microRNA in engineered M1Exos (II), M1Exos (III), M1Exos (IV). Data are presented as mean ± SD (*n* = 4, *p < 0.05, **p < 0.01, ***p < 0.001). D) Flow cytometry analysis of phenotypes of macrophages. M0 and M2 RAW264.7 cells were incubated with PBS (I) or engineered exosomes (II–IV) at concentrations of 2 µg mL^−1^ for 24 h. The surface expression of CD80, CD86, CD40, MHC II, and CD206 was analyzed. The percentage of E) CD86^+^, F) CD40^+^, G) MHC II^+^, H) CD206^+^ macrophages after treating M0 and M2 RAW264.7 cells with engineered exosomes (II–IV) at concentrations of 2 µg mL^−1^ for 24 h. Data are presented as mean ± SD (*n* = 4, *p < 0.05, **p < 0.01, ***p < 0.001). I,J) The mRNA expression level of M1 macrophage markers (*Inos*, *Tnf*, *Il6*) and M2 macrophage markers (*Il4*, *Il10*, *Fizz‐1*). I) M0 and J) M2 RAW264.7 cells were incubated with engineered exosomes (II–IV) for 24 h, followed by the quantification of the mRNA expression level by RT‐qPCR.

DDRi@CAT‐PD‐M1Exos contained lots of cargos inherited from donor M1 macrophages, which possibly could polarize and re‐polarize M0 and M2 macrophages into M1 phenotypes (**Figure** **3**A). The analysis of reverse transcription quantitative real‐time polymerase chain reaction (RT‐qPCR) demonstrated that M1 macrophage‐derived DDRi@CAT‐PD‐M1Exos contained high amounts of mRNA of M1 macrophage markers, including *Cd86*, *Il6*, *Tnf*, and *Inos* (Figure 3B, Figure S13, Supporting Information). Consistently, M2 macrophage‐derived exosomes contained high amounts of mRNA of M2 macrophage markers, including *Il4*, *Il10*, *Fizz‐1* (Figure 3B, Figure S13, Supporting Information). Previous studies had revealed dendritic cell‐derived exosomes contained functional microRNA, and after the cellular uptake of exosomes, microRNA could regulate the phenotype of the recipient dendritic cells.^[^ ^26^, ^38^ ^]^ MicroRNA also had important role in regulating the polarity of macrophage. DDRi@CAT‐PD‐M1Exos had higher expression level of miR‐155, miR‐451 compared with DDRi@CAT‐PD‐M0Exos (Figure 3C, Figure S14, Supporting Information). These microRNAs are believed to polarize M0 macrophages to M1 macrophages.^[^ ^39^ ^]^ Consistently, DDRi@CAT‐PD‐M2Exos contained higher amounts of M2‐related microRNAs (miR‐146a‐3p, miR‐34a) compared with DDRi@CAT‐PD‐M0Exos (Figure 3C, Figure S14, Supporting Information). Therefore, DDRi@CAT‐PD‐M1Exos contained various of M1 macrophage‐related mRNA and microRNA, and these cargos could possibly polarize M0 and M2 macrophages to M1 types.

To investigate the ability of engineered exosomes to polarize macrophages, RAW 264.7 cells (M0) were polarized to the M2 phenotypes by the incubation with IL‐4. M0 and M2 RAW 264.7 cells were incubated with engineered exosomes, followed by the analysis of M1 and M2 markers. The analyzed M1 markers included the surface proteins CD80, CD86, CD40, and MHC II (Figure 3D,H, Figure S15, Supporting Information), as well as the mRNA markers *Inos*, *Tnf*, and *Il6* (Figure 3I,J). The analyzed M2 markers included the surface proteins CD206 (Figure 3D,H), as well as the mRNA markers *Il4*, *Il10*, and *Fizz‐1* (Figure 3I,J). The obvious up‐regulation of M1 markers demonstrated M1 macrophage‐derived DDRi@CAT‐PD‐M1Exos could polarize M0 and M2 macrophages to M1 phenotypes. To the contrast, M2 macrophage‐derived DDRi@CAT‐PD‐M2Exos could polarize M0 macrophages to M2 phenotypes, and M0 macrophage‐derived DDRi@CAT‐PD‐M0Exos had no obvious influence on the macrophage polarization. The polarization of M2 macrophages to M1 phenotypes could possibly enhance the antitumor capability of macrophages, which was explored in following sections.

### 1.4 In vitro antitumor effects of macrophages polarized by engineered M1Exos

**Figure 4:**
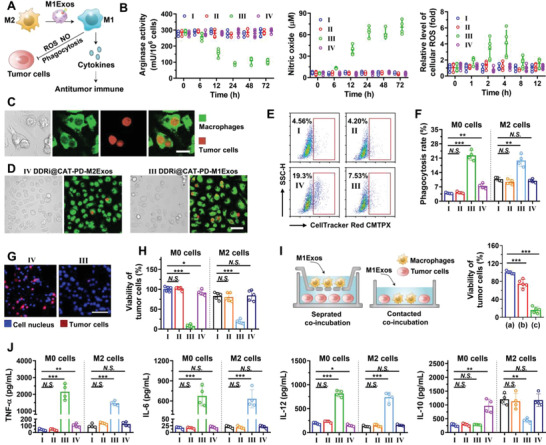
In vitro antitumor effects of macrophages polarized by engineered M1Exos. A) Schematic diagram of antitumor effects of macrophages. Engineered M1Exos could polarize M2 macrophages to M1 phenotypes, directly killing tumor cells by the generated NO, ROS and phagocytosis. The generation of lots of cytokines could possibly benefit antitumor immune in vivo. B) The arginase activity, the generation of NO and ROS of M2 RAW264.7 cells. Cells were incubated with I) PBS, II) DDRi@CAT‐PD‐M0Exos, III) DDRi@CAT‐PD‐M1Exos, IV) DDRi@CAT‐PD‐M2Exos at concentrations of 2 µg mL^−1^ for different time. C,D) The phagocytosis of macrophages was visualized by CLSM. M0 RAW264.7 cells were labeled with CellTraker Green CMFDA, and incubated with different exosomes at 2 µg mL^−1^ for 24 h. LLC cells labeled with CellTracker Red CMTPX were added and incubated for 2 h, followed by washing off LLC cells that were not phagocytosed. E,F) Flow cytometry analysis of the percentage of macrophages undergoing phagocytosis. The percentage was calculated as CellTraker Red CMTPX^+^F4/80^+^ cells in total F4/80^+^ cells. Data are presented as mean ± SD (*n* = 4, *p < 0.05, **p < 0.01, ***p < 0.001). G,H) The overall cytotoxicity of macrophages to tumor cells. After co‐incubation of RAW264.7 cells (M0 and M2), LLC cells, and engineered exosomes at concentrations of 2 µg mL^−1^ for 72 h, CLSM images were acquired for calculating the remained live LLC cells (G). LLC cells were labeled with CellTraker Red CMTPX, and the nucleus of LLC and RAW264.7 cells were stained with DAPI. I) The viability of LLC cells after the b) separated or c) contacted co‐incubation of RAW264.7 (M2), LLC cells and engineered M1Exos (2 µg mL^−1^). a) PBS was added instead of engineered M1Exos as control. J) The concentration of different cytokines in culture medium after incubating M0 or M2 RAW264.7 cells with engineered exsomes (I–IV) at concentrations of 2 µg mL^−1^ for 48 h. Data are presented as mean ± SD (*n* = 4, *p < 0.05, **p < 0.01, ***p < 0.001).

After confirming M0 and M2 macrophages could be polarized and re‐polarized to M1 phenotypes by engineered M1Exos, the in vitro antitumor effects of polarized macrophages were examined. It is believed M1 macrophages can generate nitric oxide (NO) and ROS (**Figure** **4**A), which are cytotoxic for tumor cells. However, the high expression of arginase‐1 in M2 macrophages can inhibit the generation of nitric oxide (NO), because arginase‐1 is an enzyme metabolizing L‐arginine to ornithine and polyamines, and L‐arginine is the precursor for the synthesis of NO.^[^ ^24^, ^40^, ^41^ ^]^ After incubating M2 RAW264.7 cells with DDRi@CAT‐PD‐M1Exos, the cellular arginase activity was significantly decreased by 65.1% after 24 h incubation (Figure 4B), leading to the increased generation of NO by 11.2 folds (Figure 4B). The generation of ROS was also increased by 3.2 folds after 4 h incubation (Figure 4B). However, M0Exos, DDRi@CAT‐PD‐M0Exos and DDRi@CAT‐PD‐M2Exos had little influence on the arginase activity and the generation of NO and ROS (Figure 4B and Figure S16, Supporting Information).

The phagocytosis is the important mechanism for M1 macrophages to eliminate tumor cells.^[^ ^24^ ^]^ M2 RAW264.7 cells were labeled with CellTraker Green CMFDA, and incubated with LLC cells labeled with CellTracker Red CMTPX, followed by the addition of engineered exosomes. The phagocytosis could be directly observed using CLSM (Figure 4C). The addition of engineered M1Exos obviously enhanced the phagocytosis of macrophages compared with the engineered M2Exos (Figure 4D). The percentage of macrophages undergoing phagocytosis was precisely quantified using flow cytometry (Figure 4E,F, Figure S17, Supporting Information). The phagocytosis rate of M0 cells with the addition of engineered M1Exos was 5.69 folds as high as that of the addition of PBS (Group III versus Group I for M0 cells, 22.31% versus 3.92%), and the phagocytosis rate of M2 cells with the addition of engineered M1Exos was 1.85 folds as high as that of the addition of PBS (Group III versus Group I for M2 cells, 19.86% versus 10.74%), while engineered M0Exos and M2Exos had less influence on the phagocytosis rate of macrophages (Figure 4E,F).

The overall cytotoxicity of macrophages to tumor cells was examined by the long term (72 h) co‐incubation of RAW264.7 cells (M2), LLC cells, and engineered exosomes. The minimal live LLC cells were observed after the addition of engineered M1Exos by CLSM (Figure 4G). The viability of LLC cells was largely decreased after the addition of engineered M1Exos, while engineered M0Exos and M2Exos only led to a slight decrease (Figure 4H), demonstrating M1Exos could enhance the cytotoxicity of macrophages to tumor cells.

NO generated from macrophages has a half‐life of only 4–6 s, and the generated ROS has an even shorter half‐life. Therefore, the cytotoxicity of macrophages relies on the direct contact between macrophages and tumor cells. Consistently, the separated co‐incubation of RAW264.7 (M2), LLC cells and engineered M1Exos, using the transwell chambers, led to the great decrease in cytotoxicity of macrophages, compared with the contacted co‐incubation (Figure 4I), in accordance with the previous report.^[^ ^40^ ^]^ However, after the separated co‐incubation, the viability of LLC cells was still significantly decreased by > 20% (Figure 4I), which was possibly owing to the antitumor cytokines secreted from macrophages such as TNF‐*α*.

Besides directly killing tumor cells, various of cytokines secreted from immune cells have important physiological/pathological functions in regulating antitumor immune. Therefore, the cytokines secreted from macrophages were analyzed. After incubating RAW264.7 cells with engineered exsomes for 48 h, the concentrations of the released cytokines were determined by ELISA. Engineered M1Exos led to the significantly increased secretion of TNF‐*α*, IFN‐*γ*, IL‐6 and IL‐12 by M0 and M2 RAW264.7 cells (Figure 4J, Figure S18, Supporting Information). These cytokines are markers of M1 macrophages and proinflammatory factors, which can benefit antitumor immune in vivo. To the contrast, engineered M2Exos promoted the secretion of IL‐4 and IL‐10, which are markers of M2 macrophages and anti‐inflammatory factors, hampering antitumor immune.

Therefore, as illustrated in Figure 4A, engineered M1Exos could polarize M2 macrophages to M1 phenotypes, generating antitumor effects involving: (1) the direct killing effect to tumor cells by the generated NO, ROS and direct phagocytosis; (2) the generation of a variety of proinflammatory cytokines which could benefit antitumor immune in vivo, but had slight cytotoxicity in vitro.

### 1.5 Biodistribution, Pharmacokinetics, and the ability of Engineered M1Exos to Relieve Tumor Hypoxia

**Figure 5:**
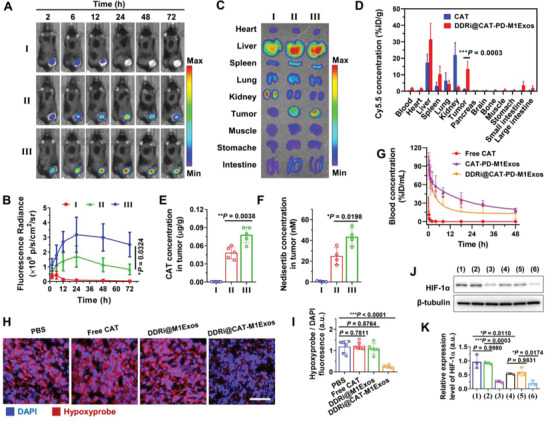
Biodistribution, pharmacokinetics, and the ability of engineered M1Exos to relieve tumor hypoxia. A) In vivo fluorescence imaging of LLC tumor‐bearing mice at different time (2–72 h) after intravenous (i.v.) administration of Cy5.5‐labeled free I) CAT, II) DDRi@CAT‐M1Exos, and III) DDRi@CAT‐PD‐M1Exos. B) Quantification of fluorescence intensity of tumor region in Figure 4A. C) Ex vivo fluorescence imaging of tumor and major organs of LLC tumor‐bearing mice. Tumor and organs were collected 6 h after the i.v. injection of Cy5.5‐labeled free CAT (I), and 24 after the i.v. injection of II) DDRi@CAT‐M1Exos and III) DDRi@CAT‐M1Exos. D) Biodistribution of Cy5.5‐labeled free CAT and DDRi@CAT‐M1Exos. Tumor and organs were collected 6 h after the i.v. injection of Cy5.5‐labeled free CAT, and 24 after the i.v. injection of DDRi@CAT‐PD‐M1Exos. E,F) The concentration of CAT and nedisertb in tumor after the i.v. injection of different agents (I–III). G) Pharmacokinetics study of Cy5.5‐labeled different agents. H,I) The degree of in vivo tumor hypoxia was examined by the staining of Hypoxyprobe‐1 kit. LLC tumors were collected 6 h after the i.v. injection of free CAT, and 24 after the i.v. injection of DDRi@M1Exos and DDRi@CAT‐M1Exos. Data are presented as mean ± SD (*n* = 6, *p < 0.05, **p < 0.01, ***p < 0.001). J) The expression of HIF‐1*α* of tumor tissue was examined by western blotting, and K) the brightness of western blotting bands was quantified by the software ImageJ for semiquantitative analysis. LLC tumors were treated with 2 Gy X‐ray irradiation 24 h after the i.v. injection of PBS or different exosomes, followed by the collection of tumors 6 h after irradiation. 1) PBS + IR; 2) DDRi@PD‐M1Exos + IR; 3) DDRi@CAT‐PD‐M1Exos + IR; 4) PBS; 5) DDRi@PD‐M1Exos; 6) DDRi@CAT‐PD‐M1Exos.

The engineered exosomes not only could polarize macrophages to enhance antitumor efficacy, but also could be utilized as drug delivery system, achieving the tumor‐targeting delivery of radiotherapy sensitizers (CAT and DDRi). The biodistribution studies were performed to evaluate the tumor‐targeting ability of the engineered exosomes. LLC tumor‐bearing mice were intravenously injected with Cy5.5‐labelled free CAT, DDRi@CAT‐M1Exos and DDRi@CAT‐PD‐M1Exos. The in vivo fluorescence imaging was monitored (**Figure** **5**A,B), the *ex vivo* fluorescence imaging of tumors and major organs was performed (Figure 5C), the biodistribution of Cy5.5 in tumors and organs was quantified (Figure 5D), and the concentration of CAT and DDRi in tumor was precisely measured (Figure 5E,F). DDRi@CAT‐M1Exos had higher accumulation in tumor compared with free CAT (Figure 5E) and free DDRi (Figure 5F), possibly resulting from the prolonged circulation time of engineered exosomes, and the passive tumor‐targeting ability derived from enhanced permeability and retention (EPR) effect. M1Exos inherited the membrane protein profile of donor macrophage cells, which possibly can actively target to the homologous macrophages in tumor.

DDRi@CAT‐PD‐M1Exos achieved higher accumulation in tumor compared with DDRi@CAT‐M1Exos, demonstrating the affinity and in vivo tumor‐targeting ability of Anti‐PD‐L1. Pharmacokinetics studies demonstrated DDRi@CAT‐PD‐M1Exos had obviously prolonged circulation time compared with free CAT (Figure 5G). The half‐life of distribution phase (t_1/2*α*_) and elimination phase (t_1/2*β*_) were increased by 8.33 and 34.75 folds, and AUC_0‐48 h_ (area under curve) was dramatically increased by 138.01 folds (Table S1, Supporting Information). CAT‐PD‐M1Exos had similar pharmacokinetic behavior as DDRi@CAT‐PD‐M1Exos, indicating the loading of DDRi had no effect on the in vivo pharmacokinetic behavior of nanocarrier.

The ability to relieve tumor hypoxia between free CAT and engineered exosomes was compared. Tumor hypoxia was examined by the immunofluorescence staining of hypoxic region of LLC tumor tissue (Figure 5H,I). Intravenously injected free CAT had negligible effect on reliving tumor hypoxia, which resulted from the short blood circulation time, lack of tumor‐targeting ability, and ease of digestion by enzymes in the blood. To the contrast, DDRi@CAT‐PD‐M1Exos could obviously relieve tumor hypoxia owing to the prolonged circulation time (Figure 5G) and enhanced tumor accumulation (Figure 5E), as well as the protection from digestion by enzymes in the blood (Figure 1N). The relief of tumor hypoxia was further examined by analyzing the expression level of the transcriptional regulator hypoxia‐inducible factor 1*α* (HIF‐1*α*), which is the key mediator of physiological and pathophysiological responses to hypoxia.^[^ ^42^ ^]^ LLC tumor‐bearing mice were divided into six groups with different treatments: (1) PBS + Irradiation (IR); (2) DDRi@PD‐M1Exos + IR; (3) DDRi@CAT‐PD‐M1Exos + IR; (4) PBS; (5) DDRi@PD‐M1Exos; (6) DDRi@CAT‐PD‐M1Exos. Tumors were treated with 2 Gy X‐ray irradiation 24 h after the i.v. injection of different exosomes, followed by the collection of tumors 6 h after irradiation for analyzing the HIF‐1*α* expression by western blotting (Figure 5J,K). The HIF‐1*α* expression of group 6 was only 33.28% of group 5, and the expression of group 3 was only 27.97% of group 2, showing the CAT loaded in DDRi@CAT‐PD‐M1Exos could obviously relieve tumor hypoxia. Besides, the HIF‐1*α* expression of group 1 was significantly higher than that of group 4 (*p = 0.0110), demonstrating radiation itself could increase the expression of HIF‐1*α* due to the reoxygenation of irradiated tumors.^[^ ^43^ ^]^

Previous studies demonstrated macrophage membrane‐coated nanomaterials had improved biocompatibility and prolonged blood circulation time.^[^ ^44^ ^]^ As the naturally occurred exosomes of macrophages with excellent compatibility, DDRi@CAT‐PD‐M1Exos also had prolonged circulation time compared with free drugs, and Anti‐PD‐L1 on exosomes further improved the tumor‐targeting ability, eventually leading to the improvement on drug efficacy. Therefore, there were various of advantages to utilize engineered M1Exos as drug delivery system.

### 1.6 In Vivo Therapeutic Efficacy of Engineered M1Exos

**Figure 6:**
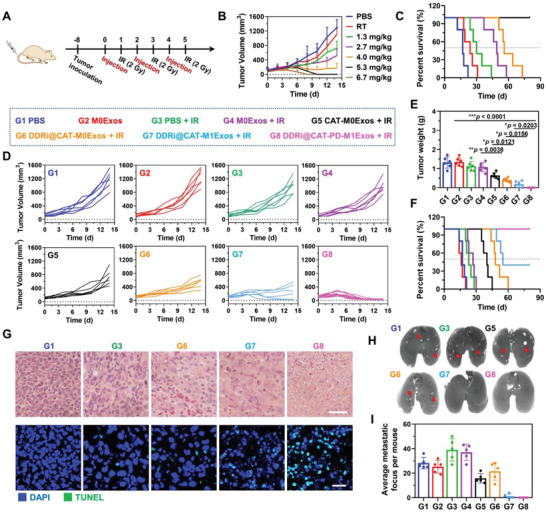
In vivo therapeutic efficacy of engineered M1Exos. A) Schedule of in vivo therapy. B,C) The therapeutic efficacy of different dosage of DDRi@CAT‐PD‐M1Exos. B) The tumor growth curve and C) mouse survival curve of LLC tumor‐bearing mice were shown. Mice were i.v. injected with different dose of DDRi@CAT‐PD‐M1Exos for three times on day 0, 2, 4, followed by 2 Gy X‐ray irradiation as shown in (A). D) The tumor growth curve, E) tumor weight, and F) mouse survival curve of LLC tumor‐bearing mice. Mice were i.v. injected with different agents (G1–G8) at 5.3 mg kg^−1^ exosomes for three times followed by irradiation (+IR) as shown in (A). A portion of mice were sacrificed for weighing tumor on day 14. G) H&E and TUNEL staining of tumor tissue collected on day 14 of in vivo therapy. Nucleus were stained with DAPI. Scale bar for H&E staining, 100 µm. Scale bar for TUNEL assay, 30 µm. H) India ink‐stained lungs collected on day 14 of in vivo therapy. The red arrows indicated the representative metastasis focus (white spots) of tumor. I) Quantification of the average number of tumor metastatic focus on lungs in (H). Data are presented as mean ± SD (*n* = 5, *p < 0.05, **p < 0.01, ***p < 0.001).

The in vivo therapeutic efficacy was evaluated on LLC tumor‐bearing mice with tumor volume of ≈100 mm^3^. Mice were intravenously injected with engineered exosomes for 3 times, followed by 2 Gy X‐ray irradiation at 24 h after each injection (therapy schedule was showed in **Figure** **6**A). The antitumor efficacy of different dosage of DDRi@CAT‐PD‐M1Exos was first studied. Higher amounts of DDRi@CAT‐PD‐M1Exos achieved better antitumor efficacy, and the dosage of 5.3 mg kg^−1^ led to the elimination of tumor without relapse up to three months (Figure 6B,C). Even the highest dosage of engineered M1Exos did not induce obvious body weight change (Figure S19A, Supporting Information), exhibiting the good in vivo biocompatibility of engineered M1Exos.

For comparing the antitumor efficacy of different components of DDRi@CAT‐PD‐M1Exos, LLC tumor‐bearing mice were divided into eight groups with different treatments: (G1) PBS; (G2) M0Exos; (G3) PBS + IR; (G4) M0Exos + IR; (G5) CAT‐M0Exos + IR; (G6) DDRi@CAT‐M0Exos + IR; (G7) DDRi@CAT‐M1Exos + IR; (G8) DDRi@CAT‐PD‐M1Exos + IR. The dosage of 5.3 mg kg^−1^ was adopted, and administration schedule was showed in Figure 6A. M0Exos had no influence on tumor growth (Figure 6D,E), exhibiting the good biocompatibility. DDRi@CAT‐M0Exos + IR achieved much better antitumor efficacy than M0Exos + IR. The speed of tumor growth was inhibited (Figure 6D), and tumor weight was decreased by 62.5% measured at day 14 (Figure 6E). The median survival time of mice extended from 27 to 48 d with significant difference (***p < 0.001). Hematoxylin and eosin (H&E) staining of tumor section showed obviously increased necrosis (Figure 6G), and terminal‐deoxynucleoitidyl transferase mediated nick end labeling (TUNEL) staining of tumor section showed significant enhanced cellular apoptosis (Figure 6G). All above exhibited the obvious radiotherapy sensitization effect of CAT and DDRi. In order to examine the CAT function of relieve tumor hypoxia, tumors were collected 6 h after the last irradiation for examining the expression level of HIF‐1*α* by western blotting (Figure S20, Supporting Information). There was little difference between the HIF‐1*α* expression of PBS + IR and M0Exos + IR (P > 0.9999), but the HIF‐1*α* expression of CAT‐M0Exos + IR was only 22.9% of that of CAT‐M0Exos + IR with significant differences (***p = 0.0009). Therefore, the CAT loaded in DDRi@CAT‐PD‐M1Exos could effectively relieve tumor hypoxia.

There was obvious enhancement of antitumor efficacy from DDRi@CAT‐M0Exos + IR to DDRi@CAT‐M1Exos + IR (Figure 6D–G), and the survival rate of mice during 3 months was increased from 0% to 33.3% (Figure 6F), which was resulted from M1Exos enhanced antitumor efficacy by polarizing M2 macrophages to M1 types and remodeling the tumor suppressive microenvironment. Owing to the utility of Anti‐PD‐L1, all tumors were eliminated without relapse up to 3 months (Figure 6D–F). In addition, the tendency of radiotherapy to promote metastasis has received more and more attention. Although DDRi@CAT‐M0Exos achieved obvious radiotherapy sensitization effect, and tumor growth was significantly inhibited, but the lung metastasis was not largely decreased (Figure 6H,I). However, DDRi@CAT‐M1Exos and DDRi@CAT‐PD‐M1Exos largely inhibited the lung metastasis, and no obvious lung metastatic focus was observed for DDRi@CAT‐PD‐M1Exos. The remodeling the tumor suppressive microenvironment possibly induced the response of systemic immune and the inhibition of distant metastases. There was little change in mouse body weight (Figure S9B, Supporting Information), parameters of blood routine (Figure S21, Supporting Information) and blood biochemistry examination (Figure S22, Supporting Information), and histological examination of major organs (Figure S23, Supporting Information). Therefore, the therapy of engineered M1Exos + RT induced negligible side effects.

Radiation‐induced intestinal injury and pneumonitis are common side effects of radiotherapy. Given that engineered M1Exos + RT could enhance radiotherapy, and might be used in the treatment of intestine and lung cancers, the possible side effects on intestine and lung were examined. 6‐week‐old female C57BL/6 mice were divided into three groups (*n* = 4) with different treatments: (1) PBS; (2) PBS + Irradiation (IR); (3) DDRi@CAT‐PD‐M1Exos + IR. Mice were i.v. injected with 5.3 mg kg^−1^ DDRi@CAT‐PD‐M1Exos or 200 µL PBS for three times, followed by abdominal (or pectoral) precision irradiation using 2 Gy X‐ray. The ROS, myeloperoxidase (MPO), and interleukin‐1*β* (IL‐1*β*) in the intestine and lung were examined at 20 d after the final radiation for the analysis of oxidation stress and inflammatory responses. Compared with the PBS group, DDRi@CAT‐PD‐M1Exos + IR showed little influence on the expression level of ROS, MPO, and IL‐1*β* (Figure S24, Supporting Information), demonstrating engineered M1Exos + RT did not induce chronic inflammation. Masson staining of tissue section showed that the percentage and distribution of collogen fibers in the intestines and lungs with the treatment of DDRi@CAT‐PD‐M1Exos + IR had little change (Figures S25 and S26), demonstrating engineered M1Exos + RT did not cause radiation‐induced fibrosis.

The treatment efficacy of engineered M1Exos + RT was further examined on an orthotopic tumor model, which microenvironment was closer to the real physiological situation compared with subcutaneous tumor. Distant metastasis accounts for the vast majority of deaths in patients with cancer. ≈60% of metastatic breast cancer patients suffer from lung or bone metastasis in their life. Traditional treatments (such as chemotherapy, radiotherapy, and targeted therapy) have limited therapeutic efficacy, and the survival rate of these patients remains very low, leaving great need for developing potential therapeutic approaches.^[^ ^45^ ^]^ Herein, the treatment efficacy of engineered M1Exos + RT was examined on the orthotopic tumor model of breast cancer lung metastases. 6‐week‐old BALB/c mice were injected with 1 × 10^5^ 4T1 cells stably transfected with luciferase at day ‐6. Mice were randomly divided into 7 groups (*n* = 5) followed by different treatments: (G1’) PBS; (G2’) PBS + IR; (G3’) M0Exos + IR; (G4’) CAT‐M0Exos + IR; (G5’) DDRi@CAT‐M0Exos + IR; (G6’) DDRi@CAT‐M1Exos + IR; (G7’) DDRi@CAT‐PD‐M1Exos + IR. Mice were *i.v*. injected with 5.3 mg kg^−1^ engineered exosomes at day 0, 2, and 4, respectively, followed by abdominal precision irradiation using 2 Gy X‐ray at 24 h after each injection (Figure S27A, Supporting Information). The tumor growth was monitored by bioluminescence imaging (Figure S27B–C, Supporting Information). G5’ could better inhibit tumor growth than G4’ and G3′, exhibiting the obvious radiotherapy sensitization effect of CAT and DDRi. The higher inhibition rate of G6’ compared with G5’ exhibited the increased therapeutic efficacy resulted from the polarization of macrophages. G7’ achieved the highest antitumor effect owing to the utility of anti‐PD‐L1 antibodies. The treatment of DDRi@CAT‐PD‐M1Exos + IR had little influence of mouse body weight (Figure S27D, Supporting Information). These results demonstrated engineered M1Exos + RT could be employed as an effective and safe treatment for breast cancer lung metastases.

### 1.7 Immune Responses Induced by Engineered M1Exos

**Figure 7:**
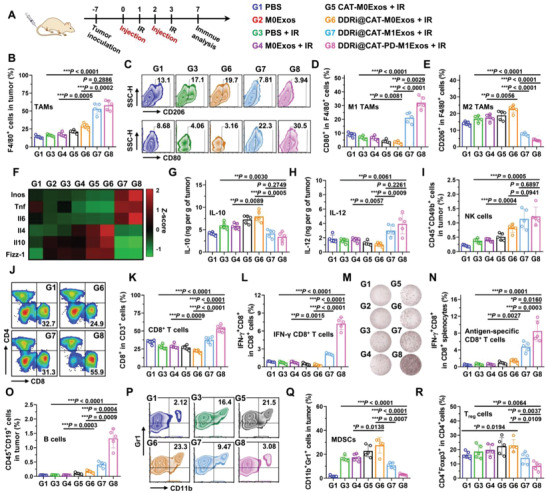
Immune responses induced by engineered M1Exos. A) Schedule and grouping of in vivo therapy for immune analysis. Mice were i.v. injected with different agents (G1–G8) at 5.3 mg kg^−1^ exosomes for two times followed by 2 Gy X‐ray irradiation (IR). B) Flow cytometry analysis of the percentage of F4/80^+^ tumor‐infiltrated macrophages (TAMs) in tumor. Flow cytometry analysis of the percentage of D) CD80^+^ and E) CD206^+^ cells in F4/80^+^ tumor‐infiltrated macrophages (TAMs), corresponding to the percentage of M1 and M2 cells in total TAMs. F) TAMs were sorted out, and the mRNA expression level of M1 and M2 markers was determined by RT‐qPCR. The expression level of G) IL‐10 and H) IL‐12 in tumor was examined by ELISA. I) Flow cytometry analysis of the percentage of CD45^+^CD49b^+^ (NK cells) in tumor. J,K) Flow cytometry analysis of the percentage of CD8^+^ in CD3^+^ T cells. L) Flow cytometry analysis of the percentage of IFN‐*γ* ^+^CD8^+^ activated T cells in total CD8^+^ T cells. M,N) IFN‐*γ* secretion of the splenocytes isolated from the different treatment groups (G1–G8) was analyzed by the ELISPOT assay. Splenocytes were re‐stimulated with OVA peptide overnight before assay. O) Flow cytometry analysis of the percentage of CD45^+^CD19^+^ cells (B cells) in tumor. P–Q) Flow cytometry analysis of the percentage of CD11b^+^Gr1^+^ (MDSCs) in tumor. R) Flow cytometry analysis of the percentage of CD4^+^Foxp3^+^ cells (T_reg_ cells) in CD4^+^ T cells. Data are presented as mean ± SD (*n* = 5, *p < 0.05, **p < 0.01, ***p < 0.001).

To facilitate the analysis of antigen‐specific immune responses, LLC^OVA+^ cells with stable expression of ovalbumin (OVA) antigen was used. LLC^OVA+^ tumor‐bearing mice were divided into eight groups with different treatments: (G1) PBS; (G2) M0Exos; (G3) IR; (G4) M0Exos + IR; (G5) CAT‐M0Exos + IR; (G6) DDRi@CAT‐M0Exos + IR; (G7) DDRi@CAT‐M1Exos + IR; (G8) DDRi@CAT‐PD‐M1Exos + IR. The injection was performed for 2 times, followed by 2 Gy X‐ray irradiation at 24 h after each injection (therapy schedule was showed in **Figure** **7**A). Tumors were collected at day 7 for immune analysis. Flow cytometry analysis showed the percentage of tumor‐associated macrophages (TAMs) of DDRi@CAT‐M0Exos + IR significantly increased compared with M0Exos + IR (***p = 0.005) (Figure 7B), demonstrating the radiotherapy sensitization effect could enhance radiotherapy, leading to the inflammatory tumor microenvironment and the recruitment of TAMs. The percentages of M1/M2 TAMs were detailedly determined using four methods: 1) M1 and M2 TAMs were identified as F4/80^+^CD80^+^ and F4/80^+^CD206^+^ cells, and the percentages were analyzed using flow cytometry (Figure 7C–E); 2) TAMs (F4/80^+^ cells) were sorted out by flow cytometry sorting, and the mRNA expression profiles of M1/M2 markers were quantified by qRT‐PCR (Figure 7F); 3) The concentration of IL‐10 and IL‐12 in tumor were determined by ELISA as the markers of M1 and M2 TAMs (Figure 7G–H); 4) The protein expression levels of M2 markers (including CD206, IL‐4, and IL‐6) and M1 markers (CD86, IL‐6, and TNF‐*α*) were visualized using immunofluorescence staining of frozen tumor tissue section (Figures S28–S33, Supporting Information). The therapeutic effect of PBS + IR, as well as the radiotherapy sensitization effect of DDRi@CAT‐M0Exos + IR, led to the increase of M2 TAMs and the decrease of M1 TAMs (Figure 7D–H), demonstrating the suppressive tumor immune microenvironment induced by radiotherapy. To the contrast, DDRi@CAT‐M1Exos + IR not only largely increased the total percentage of TAMs in tumor compared with DDRi@CAT‐M0Exos + IR (Figure 7B), but also led to the increase of M1 TAMs and the decrease of M2 TAMs (Figure 7D‐H), demonstrating the ability of M1Exos to polarize M2 TAMs to M1 phenotypes, and remodel the suppressive tumor immune microenvironment.

Besides macrophages, DDRi@CAT‐M1Exos + IR increased the percentage of tumor‐infiltrated natural killer (NK) cells (CD45^+^CD49b^+^ cells) by 4.95 folds compared with that of PBS + IR (Figure 7I). There were also obvious changes in the adaptive immune responses. The percentage of CD8^+^ T cells (Figure 7J,K) and IFN‐*γ* ^+^CD8^+^ activated T cells (Figure 7L) was slightly decreased by the enhanced radiotherapy of DDRi@CAT‐M0Exos + IR, but largely increased by DDRi@CAT‐M1Exos + IR and DDRi@CAT‐PD‐M1Exos + IR due to the remodeling of the suppressive tumor immune microenvironment. IFN‐*γ* secretion of OVA antigen‐specific CD8^+^ T cells in splenocytes were determined using enzyme‐linked immunospot (ELISPOT) assay. DDRi@CAT‐PD‐M1Exos + IR achieved the highest percentage of OVA‐specific CD8^+^ T cells compared with other groups (Figure 7M,N), exhibiting the induction of strong systemic and antigen‐specific antitumor immune responses, which could explain the inhibition of lung metastasis.

DDRi@CAT‐PD‐M1Exos + IR also largely increased the percentage of B cells (CD45^+^CD19^+^ cells) (Figure 7O). Apart from the change of antitumor immune cells, the change of suppressive immune cells was examined. The therapeutic effect of PBS + IR, as well as the radiotherapy sensitization effect of DDRi@CAT‐M0Exos + IR, largely increased the percentage of myeloid‐derived suppressor cells (MDSCs) (CD11b^+^Gr1^+^ cells) (Figure 7P,Q) and slightly elevated the portion of regulatory T (T_reg_) cells (CD3^+^CD4^+^Foxp3^+^) (Figure 7R). On the contrary, DDRi@CAT‐M1Exos + IR and DDRi@CAT‐PD‐M1Exos + IR could decrease the percentage of MDSCs and T_reg_ cells owing to the remodeling of the suppressive tumor immune microenvironment. M2 macrophages can recruit Tregs to the tumor microenvironment through release of chemokines (CCL20 and CCL22) to this milieu, and they can also induce the differentiation of myeloid cells into MDSCs probably through release of COX‐2 to the tumor microenvironment.^[^ ^46^ ^]^ Therefore, the decrease of MDSCs and T_reg_ cells was possibly due to the reduce of M2 cells caused by the engineered M1Exos.

## 2 Conclusion

In this study, a paradigm for employing immune cell‐derived exosomes to enhance radiotherapy was exhibited. M1 macrophage‐derived exosomes (M1Exos) was engineered as effective radiotherapy sensitizers. Engineered M1Exos realized the trilogy of radiotherapy sensitization, simultaneously solving three limitations that hinder the efficacy of radiotherapy through the relief of tumor hypoxia, the inhibition of DNA damage repair, and the remodeling of immunosuppressive microenvironment.

The expressed catalase in engineered M1Exos led to the relief of tumor hypoxia, and the small‐molecular inhibitor loaded in engineered M1Exos caused the inhibition of DNA damage repair. In addition, the natural characteristics of M1Exos made it possible to polarize M2 macrophages into M1 phenotypes, enhance the cytotoxicity of macrophages toward tumor cells in vitro, and promote macrophages to secrete various of cytokines beneficial for antitumor immune. The remodeling of the suppressive immune microenvironment of tumor was eventually achieved owing to the natural characteristics of M1Exos, as well as the anti‐PD‐L1 antibody expressed on engineered M1Exos for the immune checkpoint blockade. As a consequence, antitumor immune cells (M1 TAMs, NK cells, B cells, CD8^+^ T cells, activated CD8^+^ T cells, and tumor antigen‐specific CD8^+^ T cells) were increased, suppressive immune cells were down‐regulated, tumor growth and lung metastasis were thoroughly inhibited.

The excellent antitumor efficacy exhibited the great potentiality to utilize immune cell‐derived exosomes as radiotherapy sensitizers, inspiring the future efforts to explore different kinds of immune cell‐derived exosomes for enhanced radiotherapy. The superb biocompatibility and degradability, low toxicity and immunogenicity of exosomes made it promising for future clinical application.

## 3 Experimental Section

### Materials

Immnofluorescence antibodies for flow cytometry analysis of immune cells were purchased from BioLegend (USA), including APC‐anti‐mouse CD3, PE‐anti‐mouse CD4, FITC‐anti‐mouse CD8, PE/Cy7‐anti‐mouse IFN*γ*, PE‐anti‐mouse Foxp3, PE‐Cy7‐anti‐mouse CD45, APC‐anti‐mouse CD19, FITC‐anti‐mouse CD49b, PE‐anti‐mouse CD11b, APC‐anti‐mouse GR1, APC‐Cy7‐anti‐mouse F4/80, APC‐Cy7‐anti‐mouse MHC II, PE‐anti‐mouse CD40, APC‐anti‐mouse CD80, PE‐Cy7‐anti‐mouse CD86, FITC‐anti‐mouse CD206. Mouse IFN‐γ ELISA Kit (Catlog No. CZM10‐96) was purchased from Beijing CHENG ZHI KE WEI Biotechnology Co., Ltd., (China). Mouse IL‐10 and IL‐12 ELISA kit were purchased from Abcam (UK). Mouse IFN‐*γ* precoated ELISPOT kit were purchased from Dakewe Biotech Co., Ltd., (China). Anti‐c‐Myc antibody and anti‐mouse H2A.X antibody was purchased from Abcam (UK). Hypoxyprobe‐1 plus kit was purchased from Hypoxyprobe Inc., (USA). Annexin V‐FITC Apoptosis Detection Kit was purchased from BD Biosciences (USA). In situ cell death detection kit for TUNEL assay was purchased from Roche Inc. (Swiss). Nedisertib was purchased from Topscience Co. Ltd. (China). Catalase Activity Detection Kit (Ammonium Molybdate Method) was purchased from Beijing Solarbio Science & Technology Co., Ltd., (China). Reactive Oxygen Species Assay Kit was purchased from Beiing LABLEAD Trading Co., Ltd., (China). Deionized water was obtained with a Millipore Milli‐Q Gradient System (USA).

### Genetic Engineering of RAW 264.7 Cells for Stable Expression of Exogenous Fusion Protein

The sequence of exogenous fusion protein consisted of the following components from 5′ to 3′: c‐Myc tag, anti‐mouse PD‐L1 nanobody (Anti‐PD‐L1), transmembrane domain (TMD), catalase (CAT), and Flag tag. (GGGS)_3_ linkers were added between different components. The sequence of signal peptide (Signal P) for membrane localization was added to the 5’ end. The base sequence is optimized to eukaryotic preferred codons. Using restriction enzyme digestion and digation, the sequence was cloned into the EcoRI and MluI sites of lentiviral vector pSIN4‐EF1a‐LMO2‐IRES‐Puro (Addgene, USA, Plasmid #61064). The lentivirus was produced by HEK293T cells cultured in a 10 cm petri dish. HEK293T cells were transfected with pMD2.G, psPAX2 and the constructed lentiviral vector pSIN4 using Lipofectamine 2000 (Invitrogen, USA). 24 h after transfection, the medium was replaced with fresh medium with 20% FBS (fetal bovine serum) to remove the transfection reagent. After incubation for another 24 h, the medium containing the lentivirus was collected and filtered with a 0.45 µm filter (Pall, USA). The collected medium was mixed with free medium at 1: 1 ratio, and added to RAW 264.7 cells, followed by incubation for 48 h. Successfully transfected cells were screened out using 1 µg mL^−1^ puromycin (Invitrogen, USA). The expression of the exogenous fusion protein by RAW 264.7 cells was verified by flow cytometry analysis and confocal laser scanning microscopy (CLSM) (Nikon A1R+, Japan) using an anti‐c‐Myc tag antibody.

### Macrophage Polarization, Drug Loading of DDRi, Collection, and Characterization of Exosomes

RAW 264.7 cells were polarized to M1 macrophages by the incubation with 100 ng mL^−1^ lipopolysaccharide (LPS) for 48 h, or polarized to M2 macrophages by the incubation with 20 ng mL^−1^ IL‐4 for 48 h. RAW 264.7 cells were simultaneously incubated with 5 × 10^−6^ m DDRi (nedisertib) for 48 h for the drug loading of DDRi. Cells were washed and cultured in free medium for 24 h. The exosome‐contained medium was collected and successively filtered with 0.45 µm and 0.22 µm filter for removing cell debris. Exosomes were collected by ultra‐centrifugation at 150 000 g for 3 h, and washed with PBS once again. The TEM images of exosomes were acquired using a Tecnai F20 electron microscope (FEI, USA) with voltage of 200 kV after the staining of 2% uranyl acetate, and the SEM images were acquired with Helios Nanolab 600i freeze‐etching scanning electron microscope (FEI, USA). The hydrodynamic diameter distribution and the zeta potential of exosomes were measured using a dynamic light scattering system (Malvern Zetasizer Nano ZS, USA).

### Cell Culture and Animal Model

LLC cells and RAW 264.7 cells were originally obtained from American Type Culture Collection (ATCC) and confirmed by short tandem repeat analysis (STR). LLC cells and RAW 264.7 cells were cultured in DMEM medium (4.5g L^−1^ glucose) (Corning, USA) with 10% fetal bovine serum (Gibco, USA) and 100 U mL^−1^ penicillin and 100 µg mL^−1^ at 37°C in a humidified atmosphere containing 5 vol% CO_2_. LLC^OVA+^ cells with the stable expression of ovalbumin (OVA) were obtained using lentiviral transfection. Hypoxic cells were cultured in 1 vol% O_2_, 5 vol% CO_2_, and 94.9 vol% N_2_. Female C57BL/6 mice of 4–5 weeks old were obtained from Vital River Laboratory Animal Technology Co. Ltd (China). All animal experiments were approved by the Institutional Animal Care and Use Committee of National Center for Nanoscience and Technology. For establishing xenografted tumor model, 2 × 10^5^ LLC cells were dispersed in PBS and subcutaneously injected into the right hind thigh of mice.

### Analysis of Radiosensitization Effect In Vitro

The level of cellular ROS, DSB, cell apoptosis, cell viability and the colony formation assay were analyzed using LLC cells. Cells were seeded in a 24‐well plate at a density of 10^5^ cells, cultured in 20 vol% O_2_ for adherence, and further cultured under 1% O_2_ for 24 h to induce the cells into hypoxic state. The cells were incubated with different agents dissolved in 0.5 mL DMEM medium containing 10 × 10^−6^ m H_2_O_2_ for 6 h, and once again incubated with different agents dissolved in 0.5 mL DMEM medium containing 10 × 10^−6^ m H_2_O_2_, and immediately irradiated with an X‐ray radiator (Rad Source RS2000XE, USA) at a dose of 2 Gy, followed by different treatments: I) The H2DCFDA probe was used to analyze the cellular level of ROS. After X‐ray irradiation, the cells were incubated with 20 × 10^−6^ m H2DCFDA for 15 min and collected for flow cytometry analysis of the fluorescence intensity of H2DCFDA. II) Annexin V‐FITC Apoptosis Detection Kit was used for analysis of cell apoptosis. 12 h after X‐ray irradiation, adherent cells and floating cells were collected, stained with Annexin V‐FITC and PI according to the manufacturer's instructions for flow cytometry analysis. III) The immunofluorescence staining of *γ*‐H2A.X was used to analyze intracellular DSB. Cells were seeded in confocal dish (coverglass bottom dish) (Thermofisher, USA). 30 min after X‐ray irradiation, the cells were first stained with rabbit anti‐mouse *γ*‐H2A.X primary antibody (Abcam, USA) and then with FITC‐anti‐rabbit secondary antibody. Cells were stained with DAPI and observed under the CLSM. IV) The CCK‐8 was used to analyze cell viability. Cells were seeded in 96‐well plates at a density of 10^4^ cells per well and followed by same treatments as above. 24 h after X‐ray irradiation, 10 µL of CCK‐8 (Dojindo, Japan) was added to each well and incubated for 30 min, followed by the measurement of the absorbance of each well at 450 nm using a microplate reader (Biotek Epoch, USA). V) Cells were treated with different doses of X‐ray irradiation (2, 4, 6, 8 Gy). The cells were collected after irradiation, and diluted cells were seeded in petri dishes, cultured in 20 vol% O_2_ for 8 d. The cells were fixed with 4% paraformaldehyde and stained with 1% crystal violet for 5 min. The number of cell colonies in each dish was counted after wash, the survival fraction was calculated.

### Evaluation of Antitumor Efficacy In Vivo

The in vivo therapeutic efficacy was evaluated on LLC tumor‐bearing mice with tumor volume of ≈100 mm^3^. Mice were intravenously injected with different agents for 3 times. Tumor regions were irradiated with 2 Gy X‐ray using an X‐ray radiator (Rad Source RS2000XE, USA) at 24 h after each injection. Tumor volume and body weight (*n* = 6) were measured every 2 d, and tumor volume was calculated as *V* = (*L* × *W* ^2^)/2, where *L* and *W* were the length and width of the tumor, respectively. Tumor and major organs including heart, livers, spleens, lung, kidneys, stomach, intestine and brain were collected, and stained with H&E for histological examination. Tumor tissue was cryosectioned into 4 µm sections, and stained with in situ cell death detection kit for TUNEL assay. Mouse blood (*n* = 6) were sampled from eyes for blood routine examination. For analysis of lung metastasis, mice were euthanatized, lungs were perfused with 15% India ink from the upper trachea, excised and fixed in Fekete's solution as previously described.^[^ ^33^ ^]^ The metastatic nodules appeared as white focuses on the black lung surfaces.

### Tumor Digestion, Flow Cytometry Sorting, and Analysis

Tumor tissue was collected, cut into pieces, and digested with 1 mg mL^−1^ collagenase IV (Sigma‐Aldrich), 100 mg mL^−1^ DNase I (Sigma‐Aldrich) and 0.01% trypsin (Corning, USA) at 37 °C, 5% CO_2_ for 30 min. The single cell suspension was obtained using 70 µm cell strainer (BD Falcon, USA), and washed with FACS buffer (RPMI‐1640 medium containing 2% FBS and 0.1% NaN_3_). For the staining of cellular surface proteins, cells were incubated with anti‐FcR (clone: 2.4G2, BioXcell, USA) at 4 °C for 30 min to block nonspecific binding, and then stained with immnofluorescence antibodies at 4 °C for 30 min, washed with FACS buffer, and analyzed by AECE Novocyte flow cytometer (Agilent, USA). For the intranucleus staining of Foxp3, surface markers of cells (CD3^+^CD4^+^) were first stained as above, followed by the fixation and permeation using FOXP3 Fix/Perm buffer (Biolegend, USA), and the staining of PE‐anti‐mouse Foxp3. For flow cytometry sorting of TAMs, cells were stained with CD11b^+^F4^+^/80^+^, and sorted out using the FACSAria II cell sorter (BD, USA).

### Statistical Analysis

The final results were expressed as mean ± standard deviation (SD). The difference between the two groups was first analyzed by the f‐test (a similarity test of variance), and then analyzed by the two‐tailed and unpaired Student's t test. One‐way ANOVA analysis of variance was used for comparing differences between multiple groups. The two‐sided log‐rank test was used to analyze the Kaplan‐Meier curve. GraphPad Prism software was used for significance analysis. p* < 0.05, p** < 0.01 and p*** < 0.001 were considered significant.

## Conflict of Interest

The authors declare no conflict of interest.

## Author Contributions

X.L. and X.Z. conceived the project and designed the experiments. X.M., M.Y., Y.G., and Y.Y. designed and performed the experiments. X.M., Y.L., G. N., and T.Z. analyzed and discussed the data. X.L., X.Z., and X.M. wrote the paper. All authors gave approval to the final version of the manuscript.

## Supporting information

Supporting Information

## Data Availability

The data that support the findings of this study are available from the corresponding author upon reasonable request.
