# Supplementary material for: Functional Immune Cell‐Derived Exosomes Engineered for the Trilogy of Radiotherapy Sensitization
Source: Adv Sci (Weinh). 2022 Jun 17;9(23):2106031. doi: 10.1002/advs.202106031 (PMC9376809; doi:10.1002/advs.202106031)
Supplement: Supplementary file 1 — Supporting Information [file ADVS-9-2106031-s001.pdf]

ADVANCED SCIENCE
Open Access

Supporting Information

for *Adv. Sci.*, DOI 10.1002/advs.202106031

Functional Immune Cell-Derived Exosomes Engineered for the Trilogy of Radiotherapy Sensitization

Xiaotu Ma, Meinan Yao, Yu Gao, Yale Yue, Yao Li, Tianjiao Zhang, Guangjun Nie, Xiao Zhao and Xiaolong Liang**

Supporting Information

Functional immune cell-derived exosomes engineered for the trilogy of radiotherapy sensitization

Xiaotu Ma, [1, 2] *Meinan Yao,* [3] *Yu Gao,* [5] *Yale Yue,*[2] *Yao Li,* [2] *Tianjiao Zhang,* [2] *Guangjun Nie,* [2] *Xiao Zhao,*[2,4*] *and Xiaolong Liang* [1*]

[1] Department of Ultrasound, Peking University Third Hospital, Beijing 100191, P. R. China

[2] CAS Key Laboratory for Biomedical Effects of Nanomaterials and Nanosafety & CAS Center for Excellence in Nanoscience, National Center for Nanoscience and Technology of China, Beijing 100190, China

[3] Beijing Center for Disease Control and Prevention, Beijing 100013, People's Republic of China

[4] IGDB-NCNST Joint Research Center, Institute of Genetics and Developmental Biology, Chinese Academy of Sciences, Beijing, 100101, China

[5] Key Laboratory of Protein and Peptide Pharmaceuticals, CAS Center for Excellence in Biomacromolecules, Institute of Biophysics, Chinese Academy of Sciences, Beijing 100101, P. R. China

SUPPLEMENTARY METHODS

Quantitative real-time RT-PCR (qRT-PCR). Total RNA in macrophage-derived exosomes were extracted by Trizol reagent (Thermofisher, USA), and reverse-transcribed with a High-Capacity cDNA RT kit (Thermofisher, USA). Real-time PCR was performed using SYBR Green qPCR Master Mix (MedChemExpress, USA). Data were normalized to the expression level of β-Actin, and the relative expression levels of different mRNAs were calculated with $2^{-\Delta\Delta Ct}$ method. The expression levels of microRNAs were analyzed using TaqMan MicroRNA Assay Kit (Thermofisher, USA) after normalization to U6 snRNA.

Primer sequences:

Cd86 Forward: 5'-GATTATCGGAGCGCCTTTCT-3'

Cd86 Reverse: 5'-CCACACTGACTCTTCCATTCTT-3'

Tnf Forward: 5'-CCTGTAGCCCACGTCGTAGC-3'

Tnf Reverse: 5'-AGCAATGACTCCAAAGTAGACC-3'

Il4 Forward: 5'-ACTTGAGAGAGATCATCGGCATTT-3'

Il4 Reverse: 5'-AGCACCTTGGAAGCCCTACAG-3'

Il6 Forward: 5'-ACAAAGCCAGAGTCCTTCAGAGA-3'

Il6 Reverse: 5'-CTGTTAGGAGAGCATTGGAAATTG-3'

Il10 Forward: 5'-ACTGGCATGAGGATCAGCAG-3'

Il10 Reverse: 5'-CTCCTTGATTTCTGGGCCAT-3'

Fizz-1 Forward: 5'-AGGATGCCAACTTTGAATAGGA-3'

Fizz-1 Reverse: 5'-CGAGTAAGCACAGGCAGTT-3'

β-Actin Forward: 5'CGCGAGAAGATGACCCAGATC'

β-Actin Reverse: 5'-CATGAGGTAGTCAGTCAGGTCCC-3'

Fluorescence imaging, biodistribution and pharmacokinetics studies. In vivo fluorescence imaging was performed on LLC tumor-bearing mice with tumor volume of ~100 mm^3. Mice were intravenously (*i.v.*) administration with Cy5.5-labelled free CAT, DDRi@CAT-M1Vs and DDRi@CAT-PD-M1Vs. At 2, 6, 12, 24, 48, and 72 h after administration, in vivo fluorescence imaging images were acquired using the IVIS Spectrum in vivo imaging system (PerkinElmer, USA). Tumor and major organs were collected for ex vivo fluorescence imaging 6

h after the *i.v.* injection of Cy5.5-labelled free CAT, and 24 after the *i.v.* injection of DDRi@CAT-M1Vs and DDRi@CAT-M1Vs.

For the precise quantification of biodistribution, the collected tumor and major organs were weighed and homogenized with tissue homogenizer, and lysed using RIPA lysis buffer (BD, USA) and probe-ultrasonication. The fluorescence intensity of Cy5.5 was measured by a Varioskan LUX multifunctional microplate reader (ThermoFisher Scientific, USA) (λex = 678 nm, λem = 694 nm). The concentration of Cy5.5 (ID%/g) was calculated from the standard curve. The standard curves of different tissues were obtained by dissolving a gradient concentration of Cy5.5-labelled free CAT, DDRi@CAT-M1Vs or DDRi@CAT-PD-M1Vs in the corresponding tissue homogenates.

The pharmacokinetic study was performed on C57BL/6 mice of 4-5 weeks. The mice were *i.v.* injected with Cy5.5-labelled free CAT, CAT-PD-M1Vs and DDRi@CAT-PD-M1Vs. Before the injection and 15 minutes, 30 minutes, 1, 2, 4, 8, 18, 30, and 48 hours after the injection, 50 μL of blood was collected from the orbit, and immediately added to 100 μL of EDTA-2Na aqueous solution. The solution was added to 96-well plate for measuring the fluorescence intensity of Cy5.5. The plasma concentration of Cy5.5 (ID%/mL) was calculated form the standard curve. The PKslover software and the bi-compartmental model were used to calculate the pharmacokinetic parameters.

Measurement of IFN-γ-secreting CD8$^+$ T cells of splenocytes. Splenocytes were isolated from mouse spleen for intracellular IFN-γ flow cytometry analysis and ELISPOT assay. For flow cytometry analysis, splenocytes were co-cultured with OVA peptide (50 μg/mL) overnight. Splenocytes with the treatment of ionomycin (Abmole, USA) were used as positive controls. 5 h before collection, Monensin (MCE, USA) was added to splenocytes. Cells were collected for the stain of anti-CD3 and anti-CD8 antibody, followed by fixation and permeation using the fixation and permeation buffer (BioLegend, USA). Cells were further stained with anti-IFN-γ antibody before analysis on Novocyte™ Flow Cytometer (ACEA). ELISPOT assay was performed using Mouse IFN-γ precoated ELISPOT kit. Briefly, splenocytes were seeded in a 96-well plate (10^5 cells/well) with the pre-coat of anti-mouse IFN-γ antibody. After incubation with OVA peptide overnight, the secreted and captured IFN-γ was determined according to manufacturer's instruction.

Table S1. Pharmacokinetics parameters of free CAT and engineered M1Exos.

Parameter	Unit	CAT	CAT-PD-M1Exos	DDRi@ CAT-PD-M1Exos
$t_{1/2\alpha}$	h	0.12	0.67	1.12
$t_{1/2\beta}$	h	0.72	26.52	25.74
CL1	(ID%)/(ID%/g)/h	9.89	0.042	0.052
CL2	(ID%)/(ID%/g)/h	3.88	0.39	0.27
$AUC_{0\text{-}48\ h}$	ID%/g*h	10.11	1706.85	1405.37
$AUC_{0\text{-}infinite}$	ID%/g*h	10.11	2371.87	1910.03
MRT	h	0.53	37.63	35.80

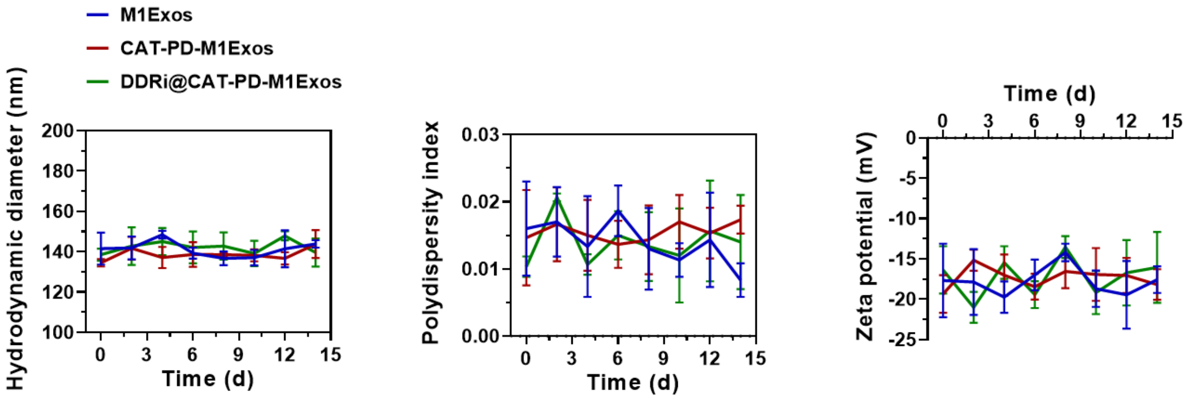

Figure S1. Stability of engineered M1Exos stored at 4 ^{o}C for 14 days, as accessed by changes of hydrodynamic diameter, polydispersity index and zeta potential.

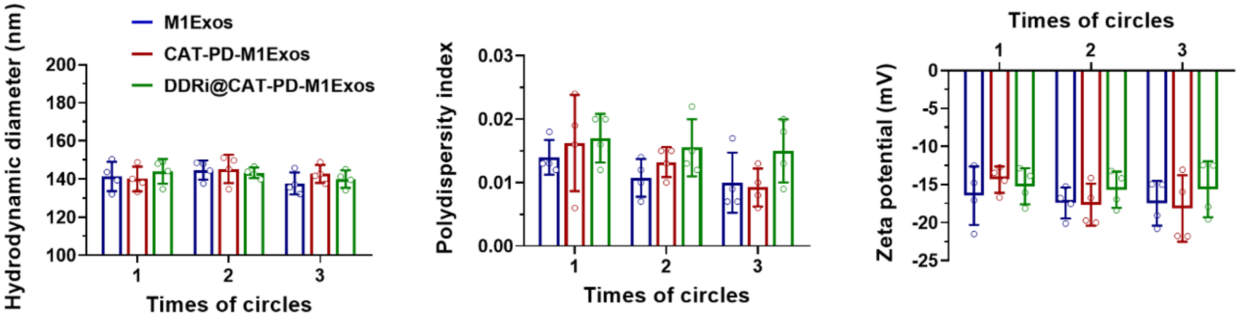

Figure S2. Stability of engineered M1Exos after three times of cycles of freezing and thawing,

as accessed by changes of hydrodynamic diameter, polydispersity index and zeta potential.

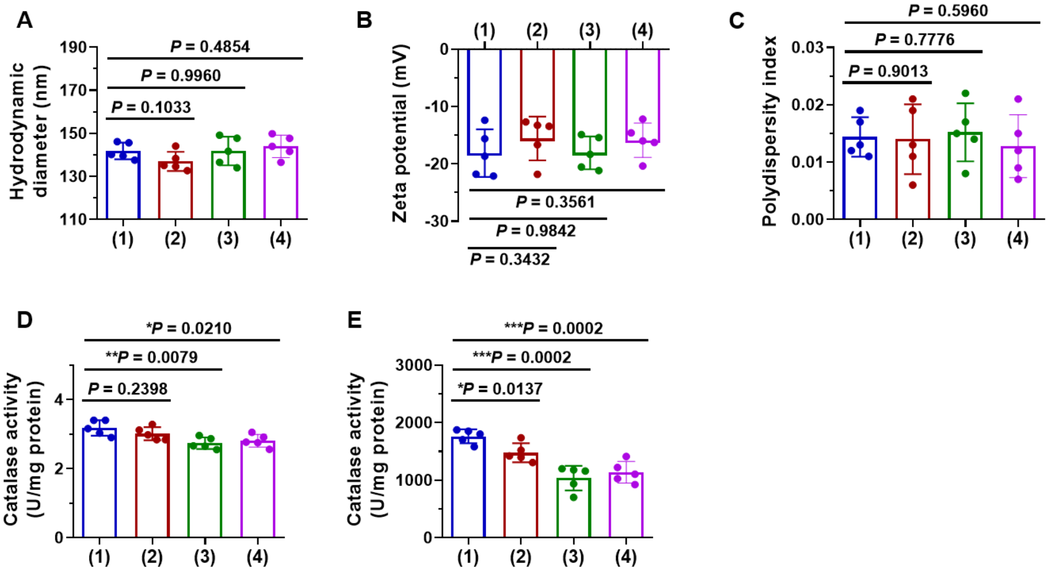

Figure S3. The stability and enzyme activity of DDRi@CAT-PD-M1Exos after the treatment of different temperature. DDRi@CAT-PD-M1Exos at the concentration of 5 μg/mL was dispersed in PBS (A-C) or fetal calf serum (D-E), followed by the incubation at different temperature for 24 h: (1) before incubation; (2) room temperature; (3) 37°C; (4) 42°C. The hydrodynamic diameter (A), zeta potential (B), polydispersity index (C) and catalase activity (D-E) of DDRi@CAT-PD-M1Exos were measured after cooling down to the room temperature. The enzyme activity of catalase was examined using Catalase Activity Detection Kit (Ammonium Molybdate Method) (Solarbio, China), and the degradation of 1 μmol of H_2O_2 per mg of protein per minute at 25°C was defined as one unit (U) of enzyme activity. The measured enzyme activity of samples was subtracted from the measured activity of the blank fetal calf serum. The catalase activity of engineered M1Exos decreased by 8.54% after the incubation in FBS at 37°C for 24 h (3.16 vs 2.89 U/mg before and after 37 ^{o}C treatment), which was possibly resulted from the degradation effect of proteases in FBS. However, the catalase activity of free catalase decreased by 40.21% after the incubation in FBS at 37°C for 24 h (1762.38 vs 1035.63 U/mg

before and after 37 °C treatment). Therefore, the membrane of engineered M1Exos could protect catalases from degradation, and increase the stability of catalase in blood.

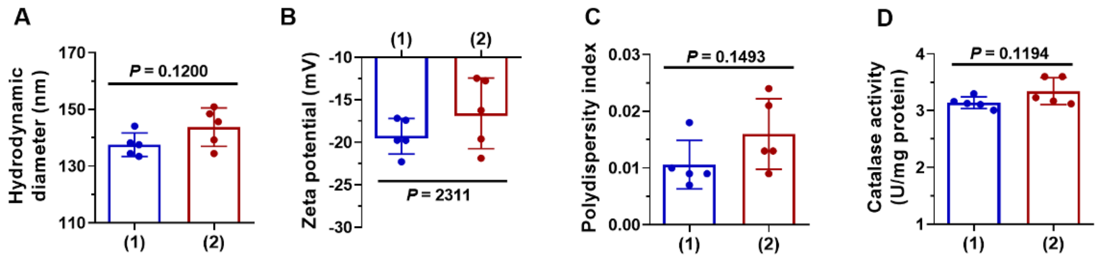

Figure S4. The stability and enzyme activity of DDRi@CAT-PD-M1Exos after the treatment of different pH values. DDRi@CAT-PD-M1Exos at the concentration of 5 μg/mL was dispersed in PBS (A-C) or fetal calf serum (D) with different pH values and kept for 24 h: (1) pH = 7.4; (2) pH = 5.4. The hydrodynamic diameter (A), zeta potential (B), polydispersity index (C) and catalase activity (D) of DDRi@CAT-PD-M1Exos were measured after adjusting the pH values back to 7.4.

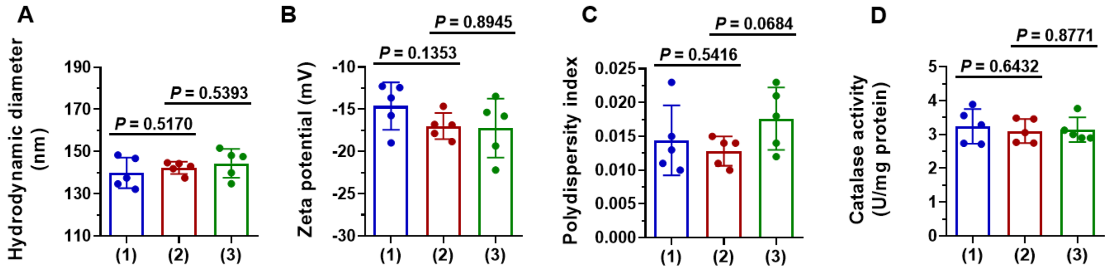

Figure S5. The change of hydrodynamic diameter (A), zeta potential (B), polydispersity index (C) and catalase activity (D) of DDRi@CAT-PD-M1Exos (5 μg/mL) before and after X-ray irradiation. (1) Before irradiation; (2) after 2 Gy irradiation; (3) after 6 Gy irradiation.

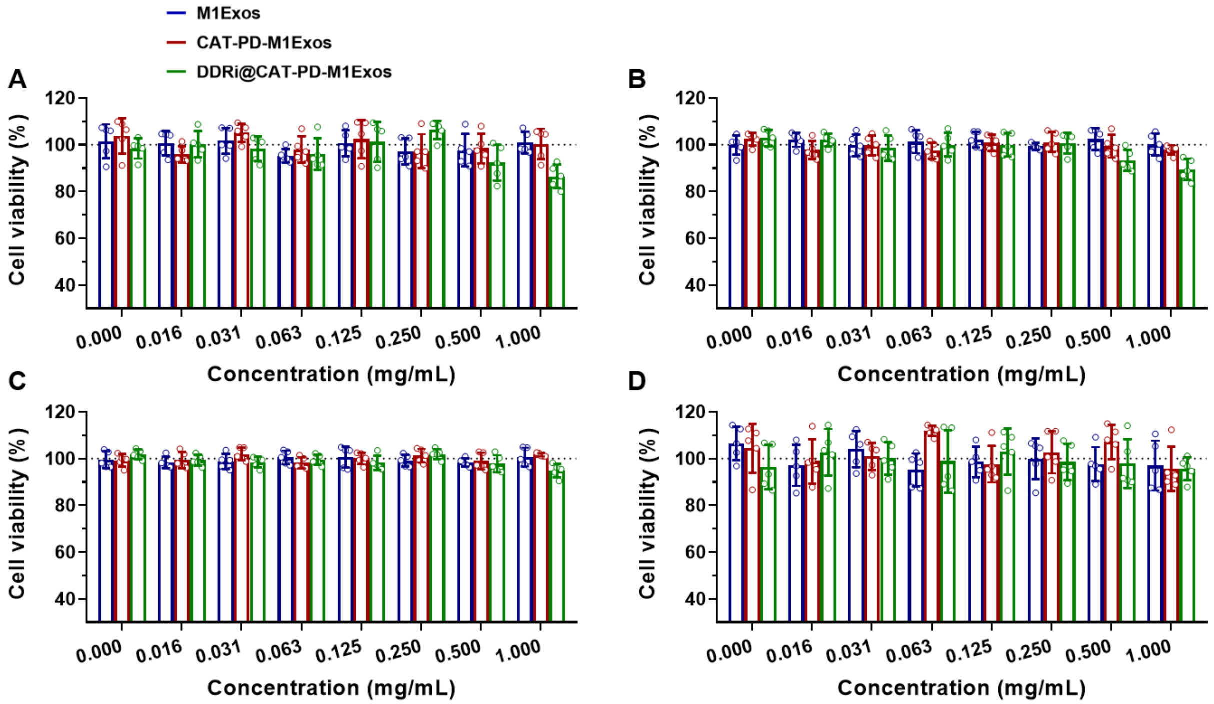

Figure S6. Cell viability after incubating with different concentrations of engineered M1Exos, exhibiting good biocompatibility of engineered M1Exos. (A) A549 human lung cancer cells; (B) LLC mouse lung cancer cells; (C) Human umbilical vein vessel endothelial cells, HUVECs; (D) 293T human embryonic kidney cells.

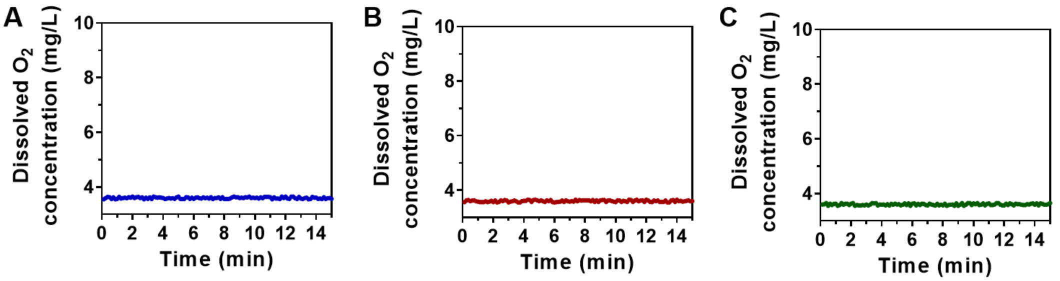

Figure S7. M1Exos (A), DDRi@M1Exos (B), and DDRi@PD-M1Exos (C) was mixed with 50 μM H_2O_2, and the concentration of dissolved oxygen was measured by on-line oxygen dissolving meter.

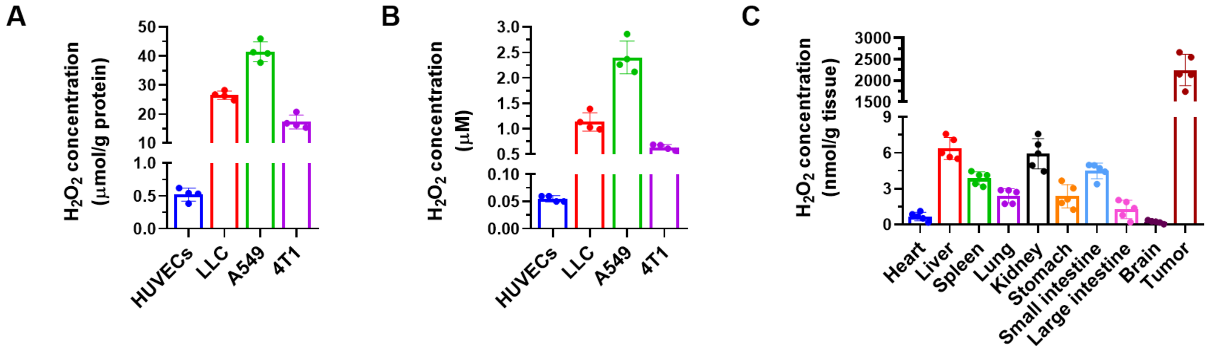

Figure S8. (A-B) The intracellular and extracellular H_2O_2 concentration of the normal cell line (HUVECs) and tumor cell lines (LLC, A549, 4T1 cells). Cells were seeded in 12-well plates and cultured for 48 h (2×10^5 cells/well) in 500 μL culture medium. Cells and culture medium were respectively collected for the determination of intracellular and extracellular H_2O_2 concentration using Hydrogen Peroxide Assay Kit (catalog # ab102500, Abcam, USA) according to the manufacturer's protocol. The amount of cellular protein was quantified using BCA Protein Assay Kit (Solarbio, China), and the H_2O_2 concentrations of different cells were normalization to the amounts of cellular proteins. (C) The H_2O_2 concentrations in LLC tumors and major organs were also determined using Hydrogen Peroxide Assay Kit according to the manufacturer's protocol. Briefly, tumor and organ tissues were collected from 6-week-old C57BL/6 mice bearing LLC tumors (~ 260 mm^3). Tissues were weighed, washed with 10 mM cold phosphate-buffered saline (pH = 7.4), homogenized in assay buffer with a Dounce homogenizer sitting on ice, and centrifuged to remove insoluble material. The H_2O_2 concentration in homogenates was determined after the performance of deproteinization. The H_2O_2 concentrations of different tissues were normalization to the weight of tissues.

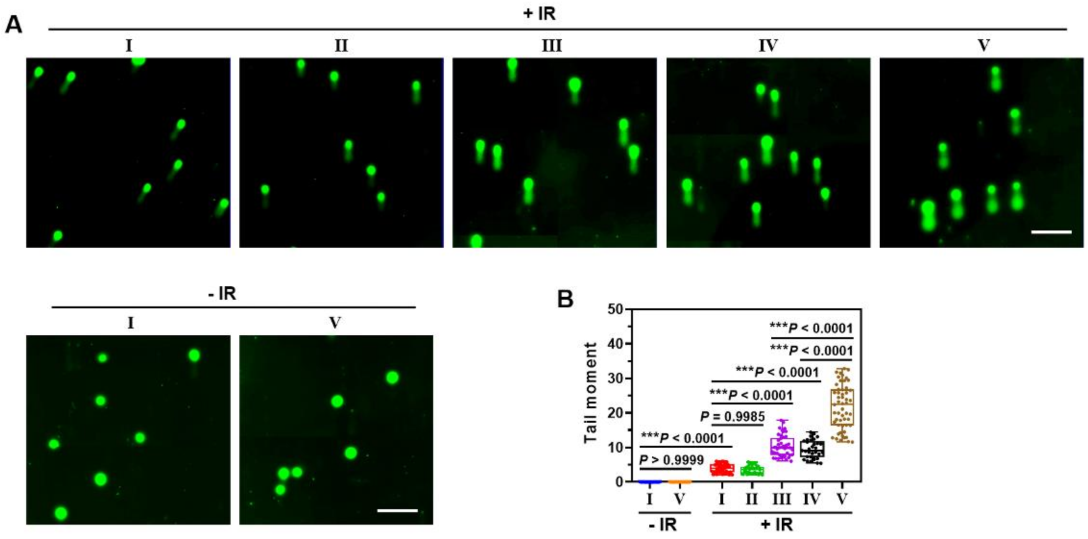

Figure S9. (A) Representative laser scanning confocal microscopy (CLSM) images of alkaline comet assay for examining the level of DNA break of LLC cells with different treatments: (I) PBS; (II) M1Exos; (III) CAT-PD-M1Exos; (IV) DDRi@M1Exos; (V) DDRi@CAT-PD-M1Exos. LLC cells were seeded into 6-well plates at a density of 10^5 cells per well and cultured for 6 h allowing the cell adherence, followed by maintaining cells in hypoxia condition (1 vol.% O_2, 5 vol.% CO_2, 94 vol.% N_2) with 10 μM H_2O_2 for 12 h to induce cells into hypoxia state. After the addition of N_2-saturated PBS (I) or exosomes (II-V) at 2 μg/mL (the concentrations of total proteins in exosomes determined by BCA protein quantification method) and incubated for 30 min, cells were treated with 1.0 Gy X-ray irradiation. After further incubation for 30 min, the alkaline comet assay was performed using Comet Assay Kit (Catalog NO. ab238544, Abcam, USA) according to manufacturers' instruction. Briefly, 70 μL of the melted Comet Agarose was added onto the Comet slides to create a Base Layer. The LLC cells were trypsinized and diluted into 1×10^5 cells/mL, which were then combined with the melted Comet Agarose at 1/10 ratio (vol./vol.). Cells were lysed and DNA were denatured by immersing Comet Slides into the pre-chilled Lysis Buffer and Alkaline Solution, followed by alkaline electrophoresis at 1 volt/cm for 30 min. The DNA was stained with Vista Green DNA Dye, and slides were viewed by CLSM

using a 488 nm laser. Scale bar, 200 μm. (B) Quantification of the tail moment calculated by the comet assay software CASP.[1] The data are shown as box and whisker plot with all data points. One-way ANOVA with a Tukey multiple comparisons test was used for statistical analysis. *, $P < 0.05$; **, $P < 0.01$; ***, $P < 0.001$.

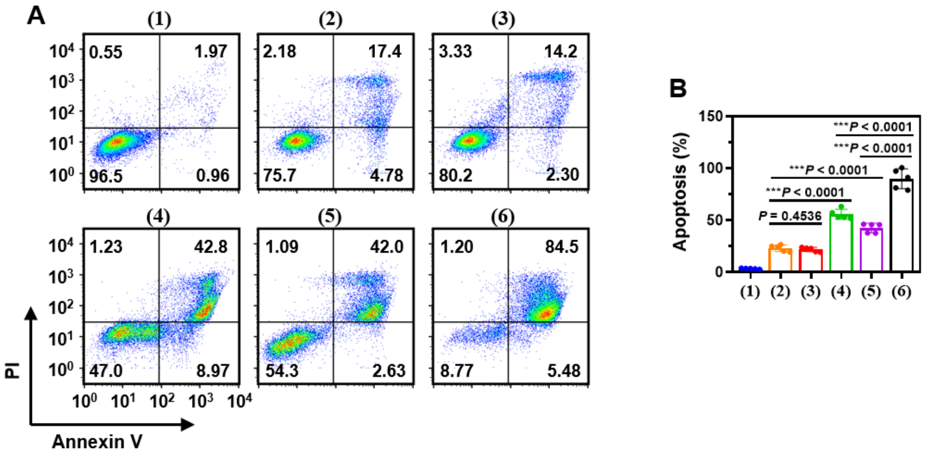

Figure S10. Flow cytometry analysis (A) and the quantification results (B) of the percentage of apoptotic 4T1 cells with/without irradiation. 4T1 cells was maintained in hypoxia condition (1 vol.% O_2) with 10 μM H_2O_2. After the addition of N_2-saturated sterile PBS or different exosomes at 2 μg/mL, cells were treated with 2 Gy X-ray irradiation (IR), followed by the Annexin V-FITC/PI double staining for examining the apoptotic cells. (1) PBS; (2) PBS + IR; (3) M1Exos + IR; (4) CAT-PD-M1Exos + IR; (5) DDRi@M1Exos + IR; (6) DDRi@CAT-PD-M1Exos + IR. The sum of Annexin V-positive and PI-positive cells in total cells were calculated as the percentage of apoptotic cells. Data are presented as mean ± SD (n = 5, *$P < 0.05$, **$P < 0.01$, ***$P < 0.001$).

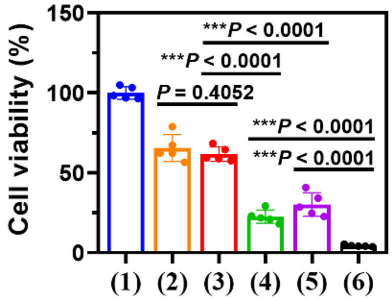

Figure 11. Cell viability of 4T1 cells with/without irradiation was examined by CCK-8 assay. (1) PBS; (2) PBS + IR; (3) M1Exos + IR; (4) CAT-PD-M1Exos + IR; (5) DDRi@M1Exos + IR; (6) DDRi@CAT-PD-M1Exos + IR. Data are presented as mean ± SD (n = 4, *P < 0.05, **P < 0.01, ***P < 0.001).

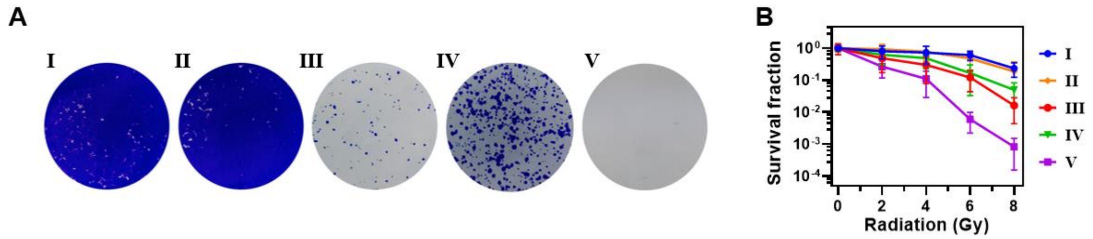

Figure 12. Colony formation assay after treating 4T1 cells with different agents (I-V) and irradiation. (I) PBS; (II) M1Exos; (III) CAT-PD-M1Exos; (IV) DDRi@M1Exos; (V) DDRi@CAT-PD-M1Exos. Photograph of cell colonies (A) and survival fraction (B) of 4T1 cells after different doses of irradiation (0-8 Gy) was shown. Data are presented as mean ± SD (n = 4, *P < 0.05, **P < 0.01, ***P < 0.001).

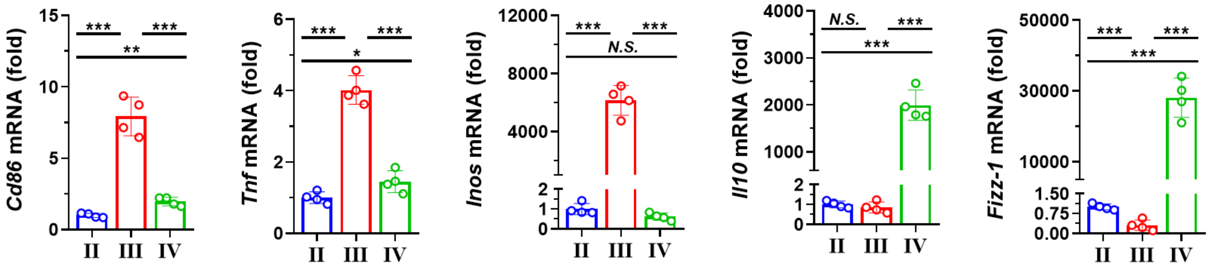

Figure S13. The relative expression level of mRNA in DDRi@CAT-PD-M0Exos (II), DDRi@CAT-PD-M1Exos (III), or DDRi@CAT-PD-M2Exos (IV).

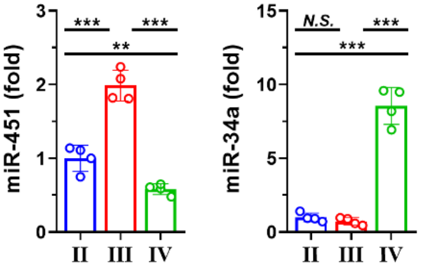

Figure S14. The relative expression level of microRNA in DDRi@CAT-PD-M0Exos (II), DDRi@CAT-PD-M1Exos (III), or DDRi@CAT-PD-M2Exos (IV).

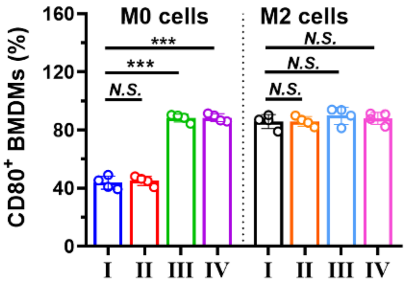

Figure S15. Flow cytometry analysis of phenotypes of macrophages. M0 and M2 RAW264.7 cells were incubated with PBS (I), DDRi@CAT-PD-M0Exos (II), DDRi@CAT-PD-M1Exos (III), or DDRi@CAT-PD-M2Exos (IV). The surface expression of CD80, CD86, CD40, MHC II, CD206 was analyzed.

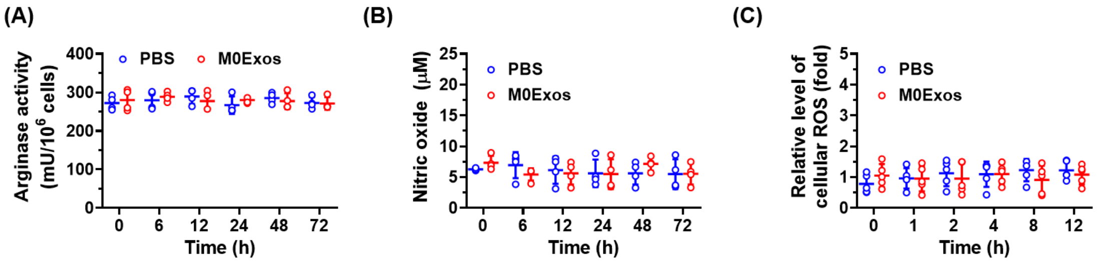

Figure S16. The arginase activity (A), the generation of NO (B) and ROS (C) of M2 RAW264.7 cells. Cells in 96-well plate (5 $\times 10^3$ per well) were incubated with 200 μL culture medium containing 10 μL PBS or M0Exos (at final concentrations of 2 μg/mL) for different time.

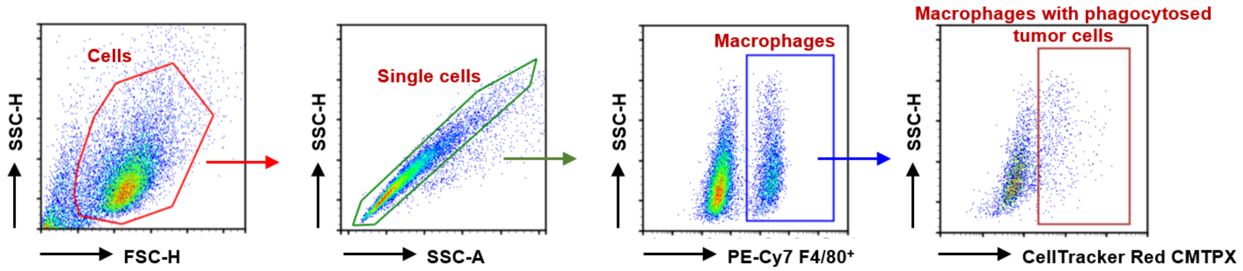

Figure S17. Gating strategy for flow cytometry analysis of the percentage of macrophages undergoing phagocytosis. The percentage was calculated as CellTraker Red CMTPX$^+$F4/80$^+$ cells in total F4/80$^+$ cells.

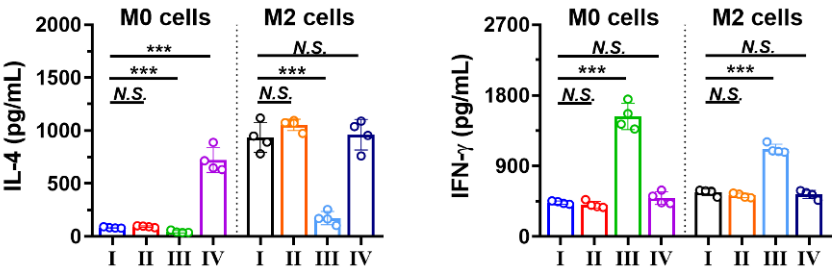

Figure S18. The concentration of different cytokines in culture medium after incubating M0 or M2 RAW264.7 cells with PBS (I), DDRi@CAT-PD-M0Exos (II), DDRi@CAT-PD-M1Exos (III), or DDRi@CAT-PD-M2Exos (IV) for 48 h.

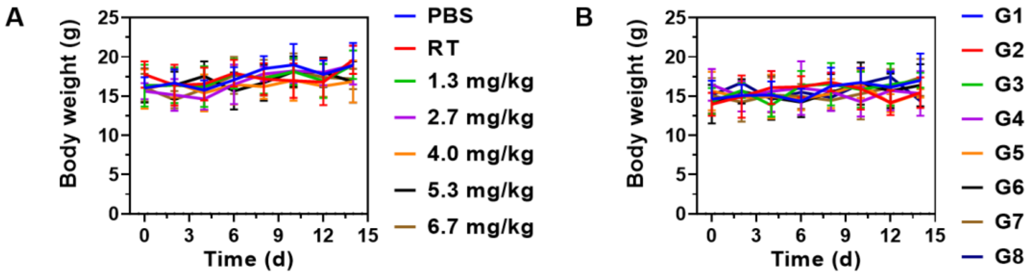

Figure S19. Changes of mouse body weight during in vivo therapy. (A) Mice were *i.v.* injected with different dose of DDRi@CAT-PD-M1Vs for three times on day 0, 2, 4, followed by 2 Gy X-ray irradiation as shown in Figure 5A. (B) Mice were *i.v.* injected with different agents (G1-G8) for three times followed by irradiation (+ IR) as shown in Figure 5A. (G1) PBS; (G2) M0Exos; (G3) IR; (G4) M0Exos + IR; (G5) CAT-M0Exos + IR; (G6) DDRi@CAT-M0Exos + IR; (G7) DDRi@CAT-M1Exos + IR; (G8) DDRi@CAT-PD-M1Exos + IR.

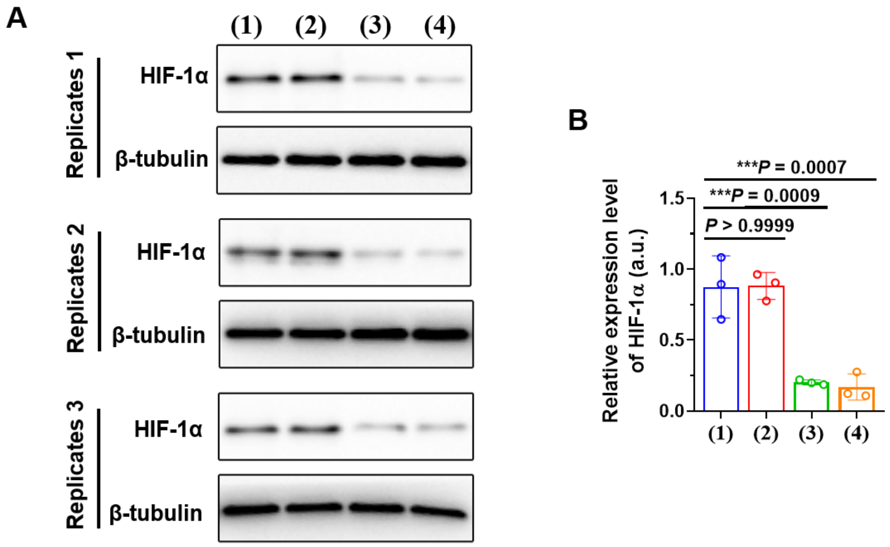

Figure S20. (A-B) The tumor expression of HIF-1α after in vivo therapy was examined by western blotting (A), and the brightness of western blotting bands was quantified by the software ImageJ for semi-quantitative analysis (B). Mice were i.v. injected with PBS or different exosomes (5.3 mg/kg) for three times on day 0, 2, 4, followed by 2 Gy X-ray irradiation at 24 h

after irradiation. Tumors were collected immediately after the last irradiation. (1) PBS + IR; (2) M0Exos + IR; (3) CAT-M0Exos + IR; (4) DDRi@CAT-PD-M1Exos + IR.

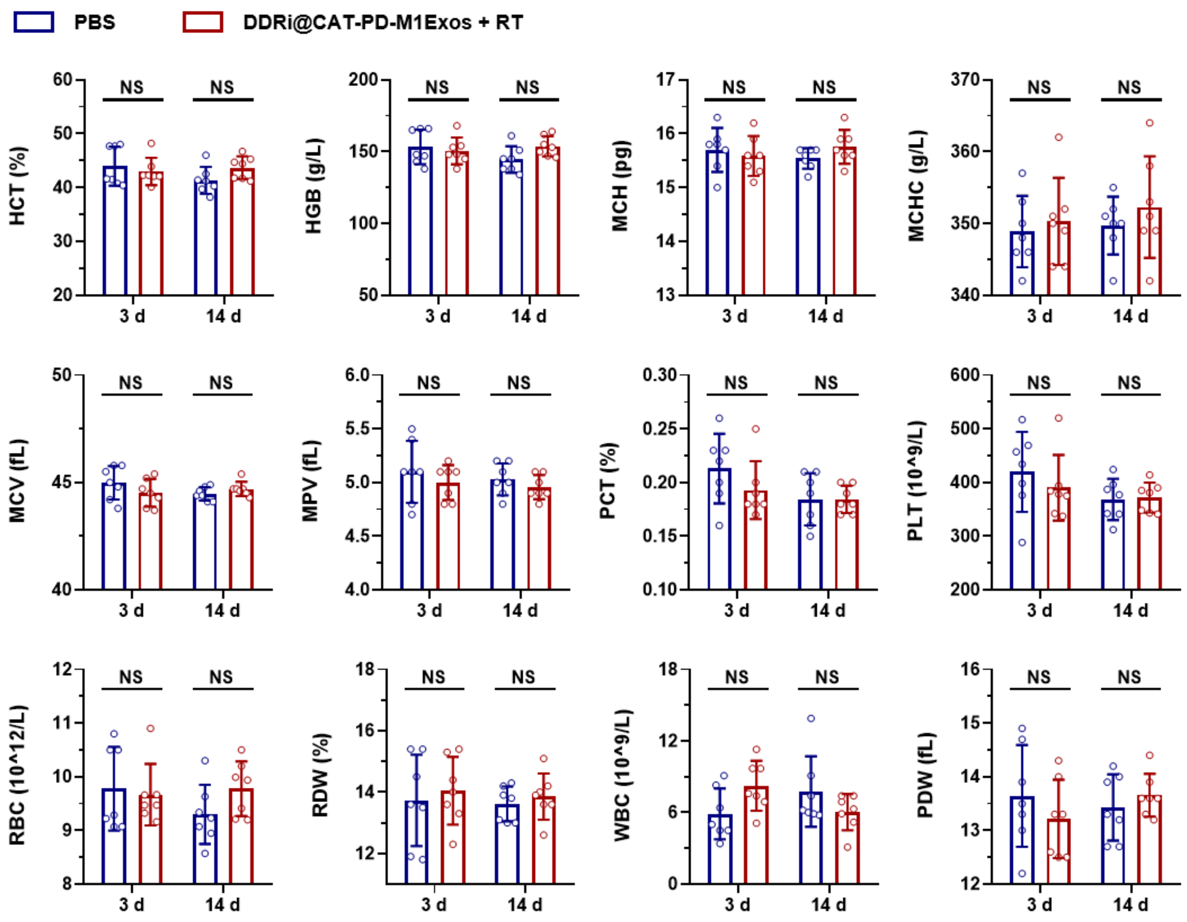

Figure S21. Parameter Changes of blood routine examination during in vivo therapy. Mice were *i.v.* injected with PBS or DDRi@CAT-PD-M1Vs for three times on day 0, 2, 4, followed by 2 Gy X-ray irradiation as shown in Figure 5A. Blood were collected on day 3 and day 14 of in vivo therapy.

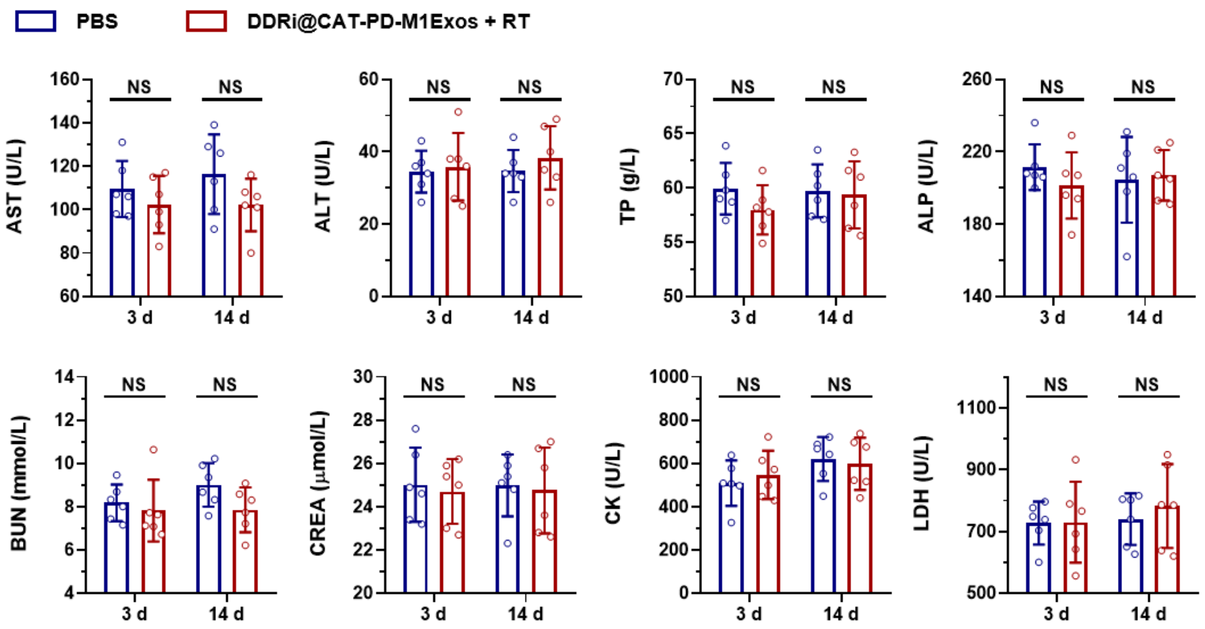

Figure S22. Changes of parameters of blood biochemistry examination during in vivo therapy. Mice were *i.v.* injected with PBS or DDRi@CAT-PD-M1Vs for three times on day 0, 2, 4, followed by 2 Gy X-ray irradiation as shown in Figure 5A. Blood were collected on day 3 and day 14 of in vivo therapy.

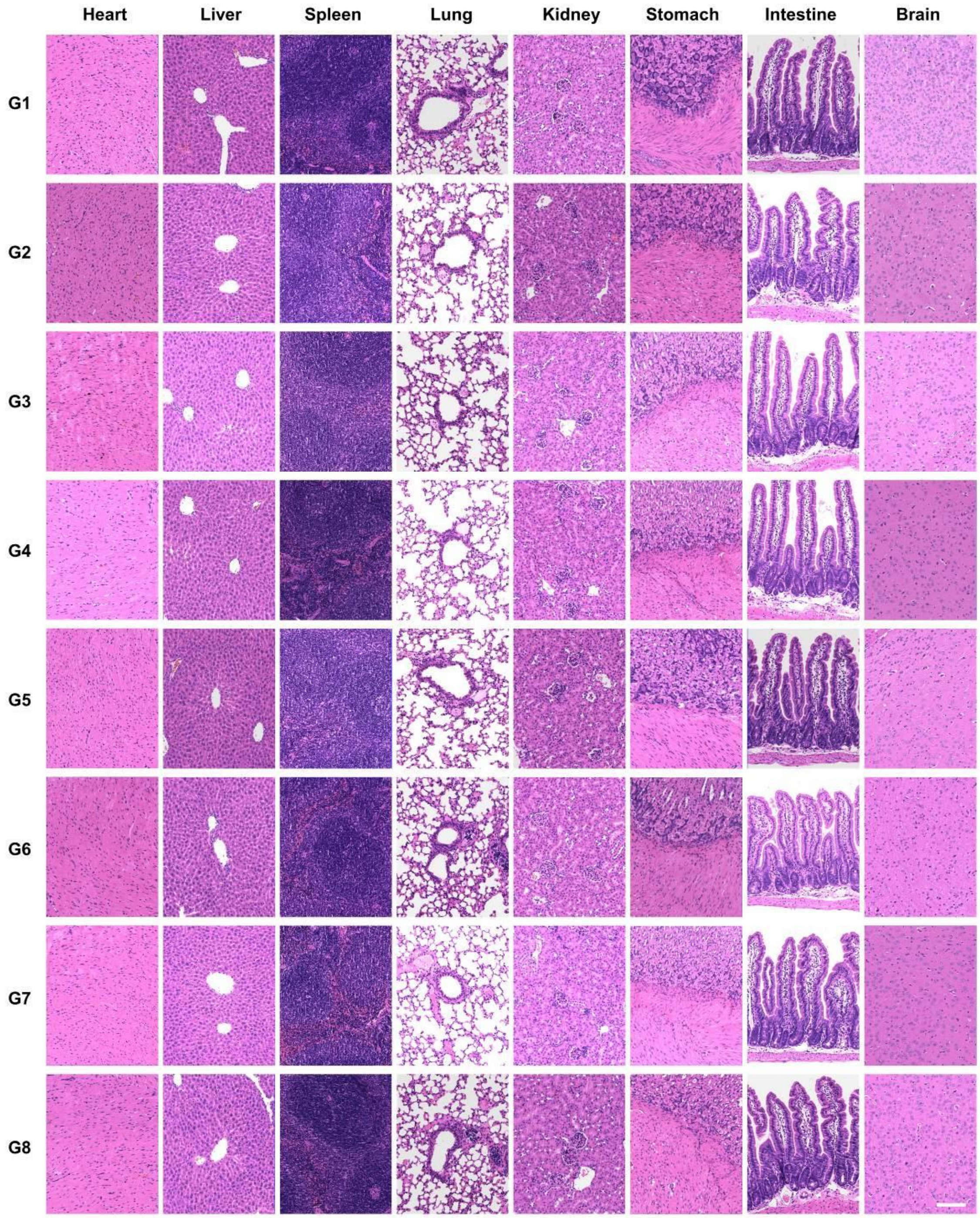

Figure S23. Histological examination of H&E-stained tissue section after in vivo therapy. Mice were *i.v.* injected with PBS (I) or DDRi@CAT-PD-M1Vs (II) for three times on day 0, 2, 4, followed by 2 Gy X-ray irradiation as shown in Figure 5A. Tissues were collected on day 14 of in vivo therapy. Scale bar, 100 μm.

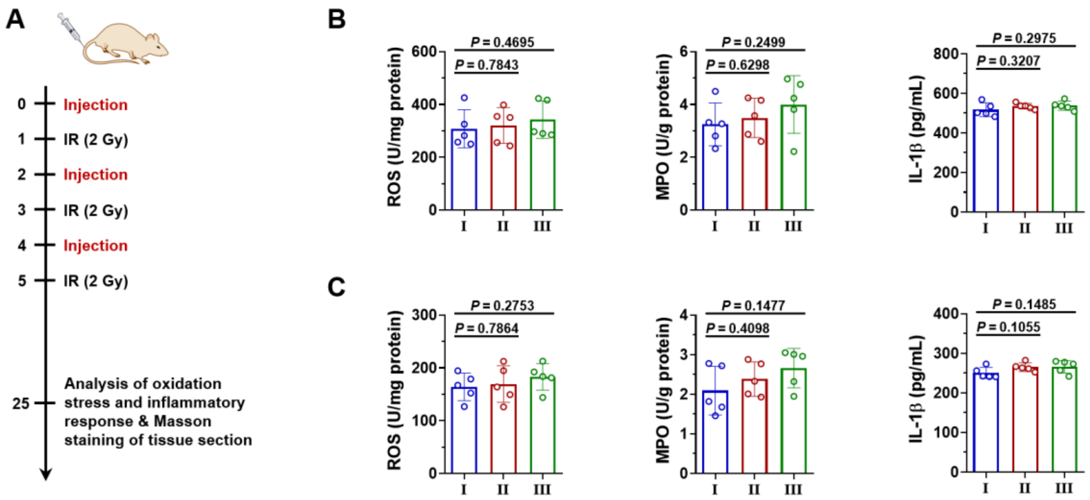

Figure S24. The analysis of oxidation stress and inflammatory responses of the intestine and lung after irradiation. (A) The schedule of examining radiation-induced side effects of intestine and lung. The 6-week-old female BALB/c mice were divided into three groups (n = 4) with different treatments: (1) PBS; (2) PBS + Irradiation (IR); (3) DDRi@CAT-PD-M1Exos + IR. Mice were i.v. injected with 5.3 mg/kg DDRi@CAT-PD-M1Exos or 200 μL PBS for three times followed by 2 Gy X-ray radiation. For evaluating side effects on intestine or lung, mouse abdomen or chest was respectively irradiated. Mice were sacrificed at 20 days after the final irradiation, and the small intestines and lungs were collected for Masson staining of tissue section, and for the analysis of oxidation stress and inflammatory responses. (B-C) Changes of the reactive oxygen species (ROS), myeloperoxidase (MPO), and interleukin-1β (IL-1β) in the intestines (B) and lungs (C) after irradiation.

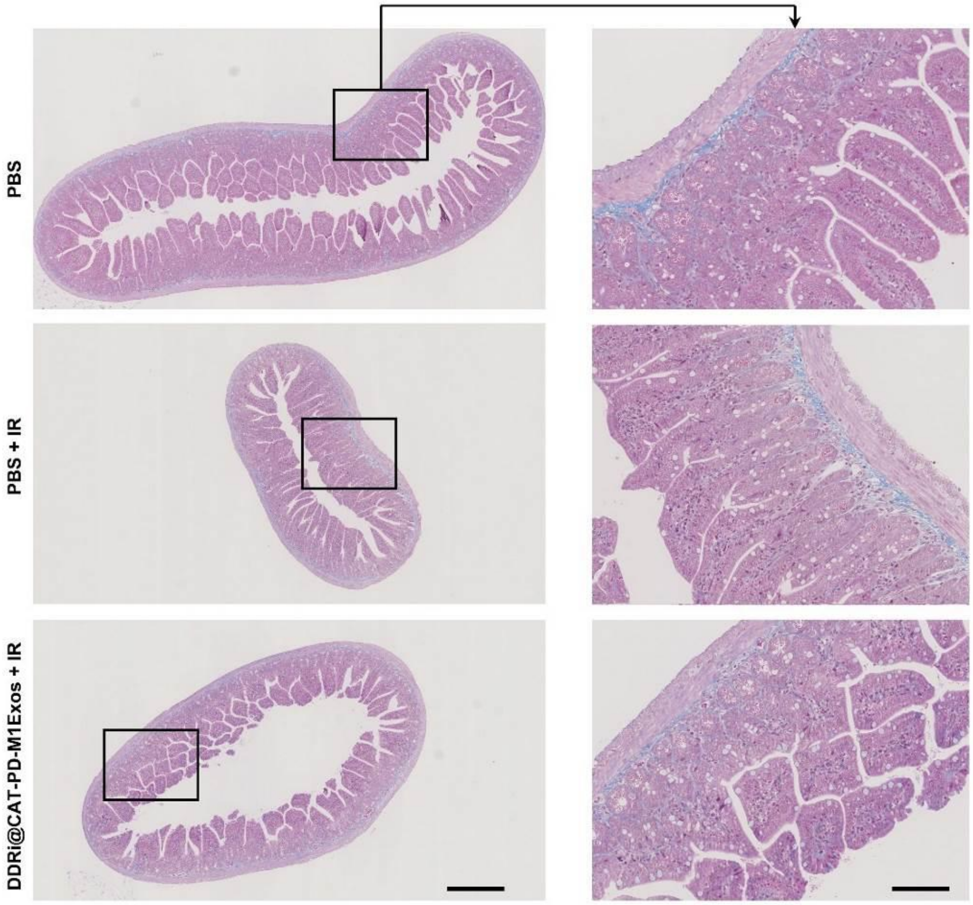

Figure S25. Masson staining of intestine sections showing the distribution of collagen in small intestines. Cells and nucleus were stained red and dark blue with ponceau and hematoxylin, while collagen fibers were stained blue with aniline blue. Scale bar for left panel, 400 μm; scale bar for right panel, 100 μm.

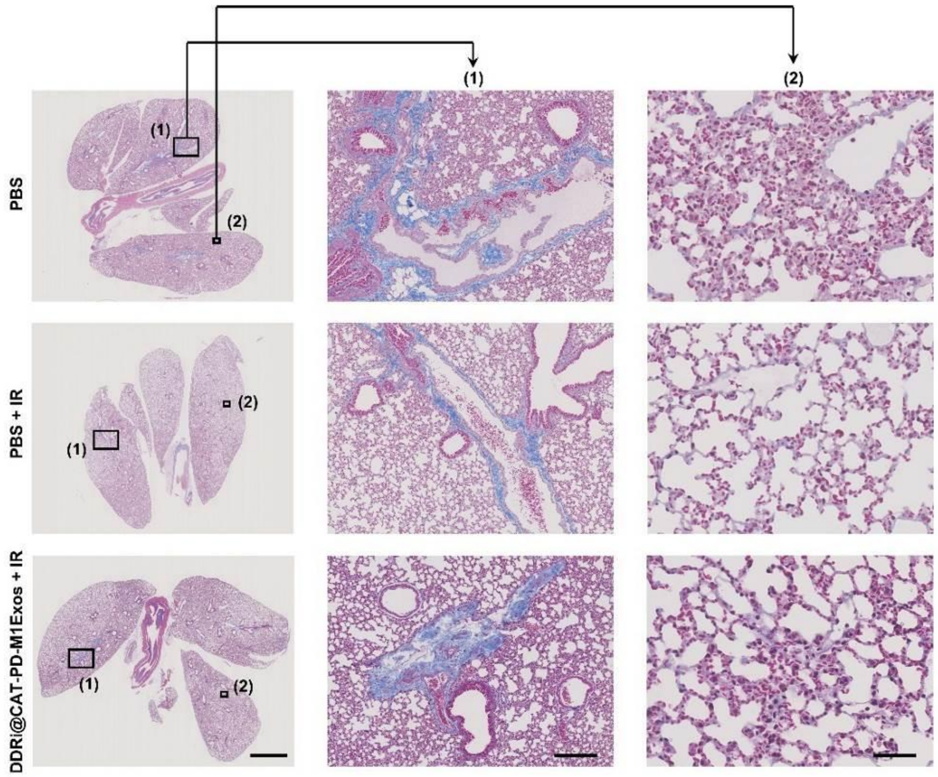

Figure S26. Masson staining of lung sections showing the distribution of collagen in lungs. Scale bar for left panel, 2 mm; scale bar for medium panel, 200 μm; scale bar for right panel, 50 μm.

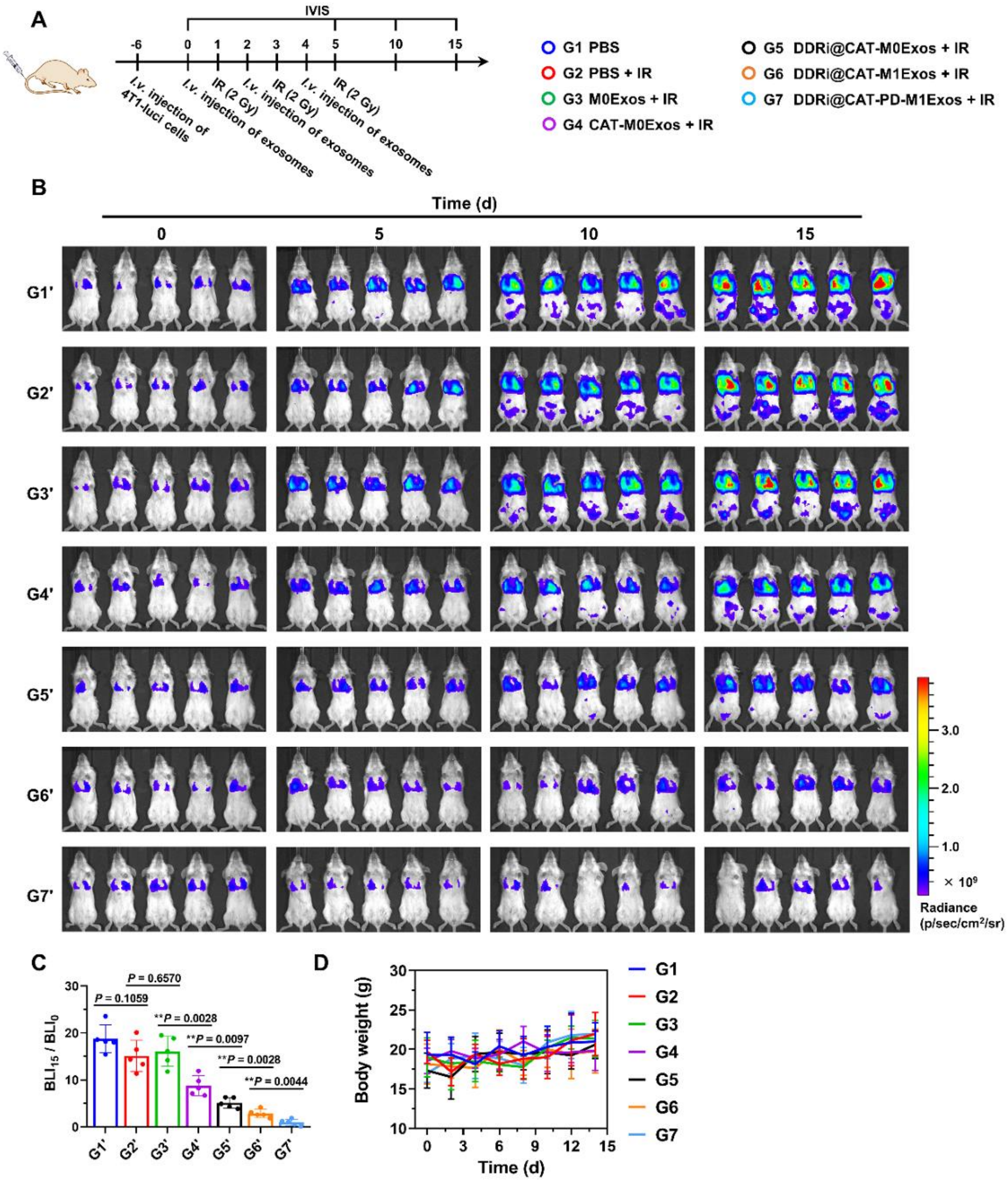

Figure S27. (A) Scheme and grouping of *in vivo* therapy using an orthotopic tumor model of breast cancer lung metastases. 6-week-old BALB/c mice were injected with 1×10^5 4T1 cells stably transfected with luciferase (4T1-luci) at day -6. Mice were *i.v.* injected with 5.3 mg/kg engineered exosomes at day 0, 2, and 4, respectively. The left and right thoracic cavities were irradiated with 2 Gy X-rays at 24 h after injection, and the spine was shielded with lead plates to

avoid irradiation. (B) The growth of orthotopic 4T1-luci tumor was monitored by bioluminescence imaging performed at day 0, 5, 10, and 15. (C) The relative growth speed of orthotopic 4T1-luci tumor was calculated by the change of bioluminescence (BLI) intensity in lung. BLI_{15} / BLI_0, the ratio of BLI intensity measured at day 15 and day 0. (D) Change of mouse body weight during *in vivo* therapy.

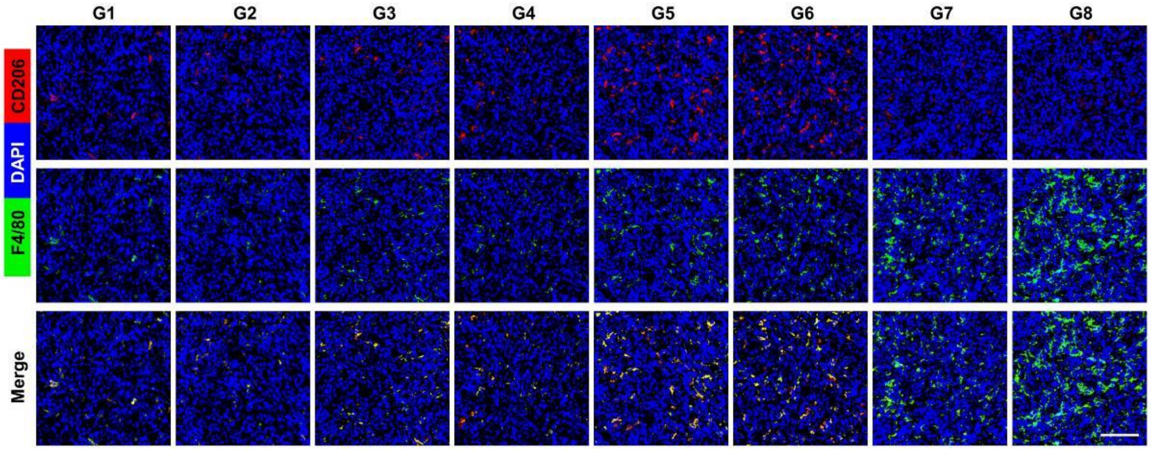

Figure S28. Immunofluorescence staining of F4/80$^+$ and CD206$^+$ cells in tumor tissue sections. Frozen sections were stained with Alexa Fluor® 488 anti-mouse F4/80 antibody (Clone: BM8. Biolegend, USA) and Alexa Fluor® 647 anti-mouse CD206 antibody (Clone: C068C2. Biolegend, USA). Cell nucleus were stained with DAPI. Scale bar, 100 μm.

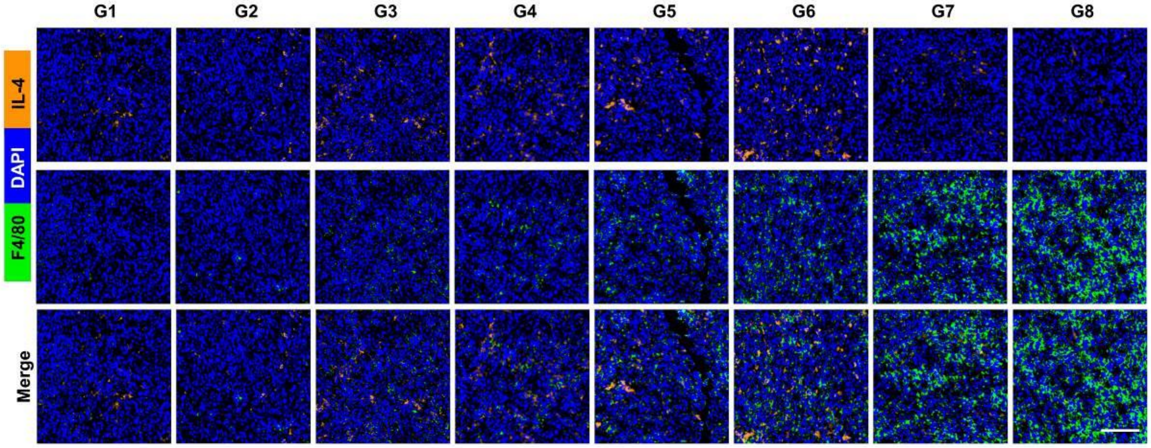

Figure S29. Immunofluorescence staining of F4/80$^+$ and IL-4$^+$ cells in tumor tissue sections. Frozen sections were stained with Alexa Fluor® 488 anti-mouse F4/80 antibody (Clone: BM8. Biolegend, USA), rabbit anti-mouse IL-4 primary antibody (Catalog # PA5-115416, Thermofisher, USA) and Alexa Fluor® 647 donkey anti-rabbit IgG second antibody (ab150075, Abcam, USA). Cell nucleus were stained with DAPI. Scale bar, 100 µm.

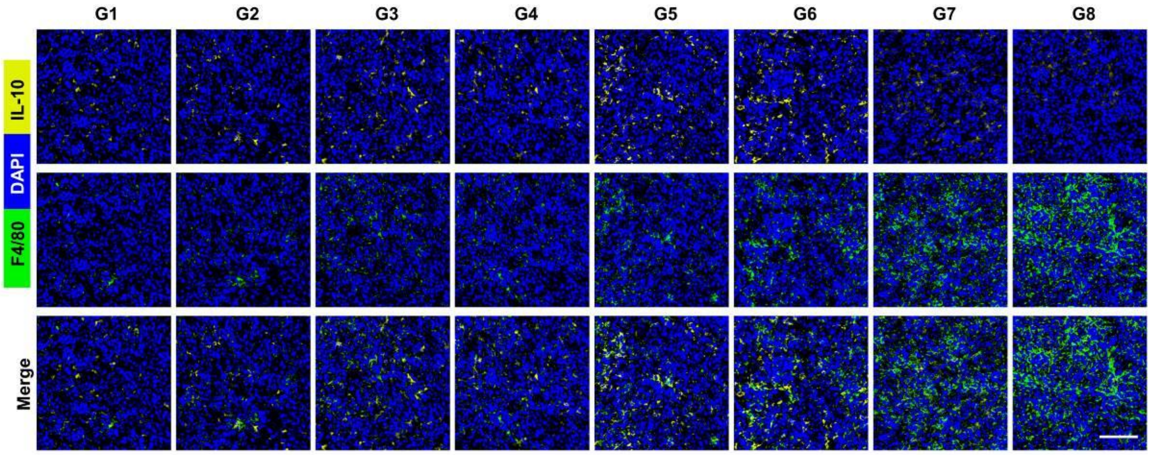

Figure S30. Immunofluorescence staining of F4/80$^+$ and IL-10$^+$ cells in tumor tissue sections. Frozen sections were stained with Alexa Fluor® 488 anti-mouse F4/80 antibody (Clone: BM8. Biolegend, USA), rabbit anti-mouse IL-10 primary antibody (Catalog # ab9969, Abcam, USA) and Alexa Fluor® 647 donkey anti-rabbit IgG second antibody (Catalog # ab150075, Abcam, USA). Cell nucleus were stained with DAPI. Scale bar, 100 µm.

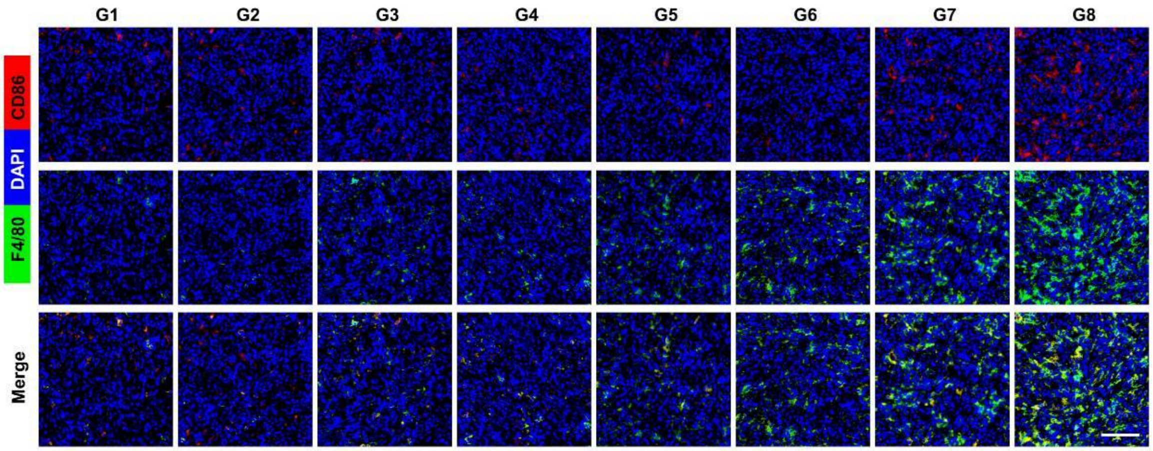

Figure S31. Immunofluorescence staining of F4/80^{+} and CD86^{+} cells in tumor tissue sections. Frozen sections were stained with Alexa Fluor® 488 anti-mouse F4/80 antibody (Clone: BM8. Biolegend, USA), rabbit anti-mouse CD86 primary antibody (Clone: EPR22958-106. Abcam, USA) and Alexa Fluor® 647 donkey anti-rabbit IgG second antibody (Catalog # ab150075, Abcam, USA). Cell nucleus were stained with DAPI. Scale bar, 100 μm.

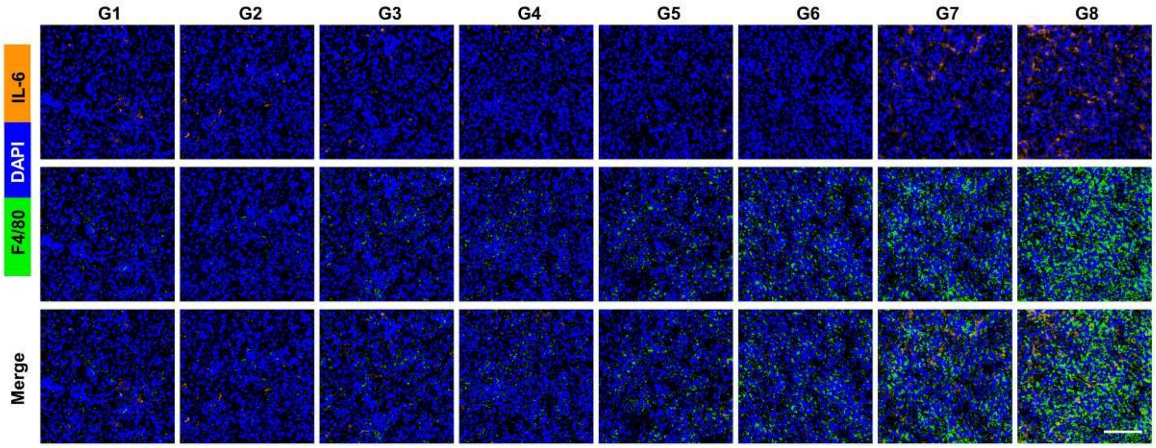

Figure S32. Immunofluorescence staining of F4/80^{+} and IL-6^{+} cells in tumor tissue sections. Frozen sections were stained with Alexa Fluor® 488 anti-mouse F4/80 antibody (Clone: BM8. Biolegend, USA), rabbit anti-mouse IL-6 primary antibody (Clone: EPR16610-69. Abcam, USA) and Alexa Fluor® 647 donkey anti-rabbit IgG second antibody (Catalog # ab150075,

Abcam, USA). Cell nucleus were stained with DAPI. Scale bar, 100 μm.

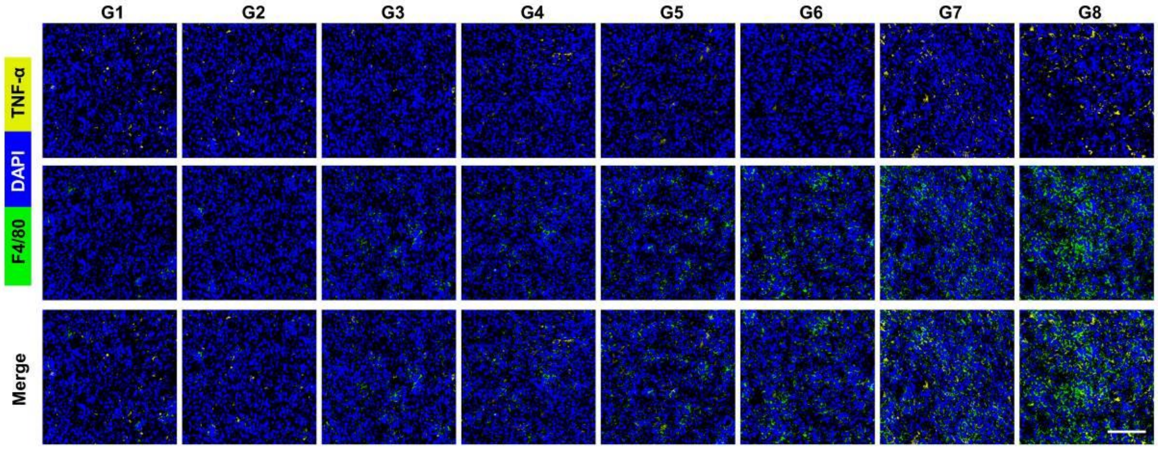

Figure S33. Immunofluorescence staining of F4/80^{+} and TNF-α^{+} cells in tumor tissue sections. Frozen sections were stained with Alexa Fluor® 488 anti-mouse F4/80 antibody (Clone: BM8. Biolegend, USA), rabbit anti-mouse TNF-α primary antibody (Clone: EPR20972. Abcam, USA) and Alexa Fluor® 647 donkey anti-rabbit IgG second antibody (Catalog # ab150075, Abcam, USA). Cell nucleus were stained with DAPI. Scale bar, 100 μm.

Reference

1. Konca, K.; Lankoff, A.; Banasik, A.; Lisowska, H.; Kuszewski, T.; Gozdz, S.; Koza, Z.; Wojcik, A., A cross-platform public domain PC image-analysis program for the comet assay. *Mutat Res* **2003,** *534* (1-2), 15-20.
